# Analyzing Stability and Change in Dyadic Attachment: The Multi-Rater Latent State-Trait Model With Autoregressive Effects

**DOI:** 10.3389/fpsyg.2021.604526

**Published:** 2021-07-01

**Authors:** Johannes Bohn, Jana Holtmann, Esther Ulitzsch, Tobias Koch, Maike Luhmann, Michael Eid

**Affiliations:** ^1^Department of Education and Psychology, Freie Universität Berlin, Berlin, Germany; ^2^Psychologische Hochschule Berlin, Berlin, Germany; ^3^Leibniz Institute for Science and Mathematics Education, Kiel, Germany; ^4^Department of Psychology, Friedrich-Schiller-Universität Jena, Jena, Germany; ^5^Department of Psychology, Ruhr University Bochum, Bochum, Germany

**Keywords:** parental attachment, latent state-trait, multitrait-multimethod, dyadic data, emerging adulthood, stability

## Abstract

Previous research suggests that parental attachment is stable throughout emerging adulthood. However, the relationships between the mutual attachments in the dyads of emerging adults and their parents are still unclear. Our study examines the stability and change in dyadic attachment. We asked 574 emerging adults and 463 parents at four occasions over 1 year about their mutual attachments. We used a latent state-trait model with autoregressive effects to estimate the time consistency of the attachments. Attachment was very stable, and earlier measurement occasions could explain more than 60% of the reliable variance. Changes of attachment over time showed an accumulation of situational effects for emerging adults but not for their parents. We estimated the correlations of the mutual attachments over time using a novel multi-rater latent state-trait model with autoregressive effects. This model showed that the mutual attachments of parents and emerging adults were moderately to highly correlated. Our model allows to separate the stable attachment from the changing attachment. The correlations between the mutual attachments were higher for the stable elements of attachment than for the changing elements of attachment. Emerging adults and their parents share a stable mutual attachment, but they do not share the changes in their respective attachments.

## Introduction

Many studies indicate that the quality of attachment to one’s parents is very stable over time (Fraley, 2002; Fraley et al., 2011; Jones et al., 2018). However, other studies are showing that the quality of attachment changes over the life course (e.g., Pinquart et al., 2013). In particular, in stressful life periods and in times of transition, attachment can change (Fraley, 2019). Due to these dynamics of attachment, the stability of attachment differs between different periods of life: While the stability of attachment is very high in childhood after the age of 6 years (Pinquart et al., 2013), it is only moderate to high in emerging adulthood (Asendorpf and Wilpers, 2000; Hiester et al., 2009; Allen et al., 2018).

According to Bowlby’s attachment theory, attachment experiences with attachment figures (in most cases, the parents) in early childhood create a working model that influences attachment in later relationships (Bowlby, 1969). Attachment is dimensional (Fraley et al., 2015), and it ranges from insecure to secure attachment (Asendorpf et al., 1997). The difference between insecure versus secure attachment is the most critical distinction in studies about attachment (Fox et al., 1991), especially in non-clinical studies where a majority is securely attached (Mikulincer and Shaver, 2016). In more clinical samples, the insecure pole of the attachment dimension can be further divided into more anxious insecurity and more avoidant insecurity (Mikulincer and Shaver, 2016).

In adolescence and emerging adulthood, the number of potential attachment figures (like parents, friends, romantic partners, or siblings) grows. Emerging adulthood is a period with many changes in lifestyle and normative expectations (Arnett, 2000), which have different effects on different relationships. However, many studies investigating attachment in emerging adulthood examine a global attachment style (e.g., Bartholomew and Horowitz, 1991; Shaver and Brennan, 1992; Scharfe and Bartholomew, 1994; Baldwin and Fehr, 1995; Davila et al., 1997; Lopez and Gormley, 2002; Scharfe and Cole, 2006; Allen et al., 2018). The global attachment style describes the tendency of persons to behave similarly in relationships with different figures. Global attachment styles are of medium stability (Scharfe and Bartholomew, 1994; Baldwin and Fehr, 1995; Lopez and Gormley, 2002). Stability is higher in times of stressful life events like breakups (Scharfe and Cole, 2006). An important limitation of studies on global attachment style is that they neglect that attachment is at least partially specific to a specific attachment figure (Bowlby, 1969; Ainsworth, 1989). Therefore, the stability and change of attachment to specific attachment figures should be examined.

In childhood, parents are the most influential attachment figures, and parental attachment remains important in emerging adulthood. Many emerging adults leave their parents’ homes, which can improve the quality of attachment between the parents and the emerging adult (Golish, 2000), especially for males (Hiester et al., 2009). A secure attachment to important figures (like parents, friends, and romantic partners) is related to various positive life outcomes in emerging adulthood such as higher well-being (La Guardia et al., 2000; Karreman and Vingerhoets, 2012), lower levels of distress (Caron et al., 2012), and better college adjustment (Lopez and Gormley, 2002; Hiester et al., 2009). Given its influence on relevant life outcomes, the analysis of stability and change of parental attachment in emerging adulthood is important.

The examination of attachment over time was limited in many studies because these studies focused on the view of one person in each dyad (e.g., Kirkpatrick and Hazan, 1994; Scharfe and Bartholomew, 1994; Baldwin and Fehr, 1995; Lopez and Gormley, 2002; Scharfe and Cole, 2006; Hiester et al., 2009; Fraley et al., 2011; De Goede et al., 2012; Jones et al., 2018). However, in a relationship-specific view of attachment, attachment has a strong dyadic component. In order to understand attachment in dyadic relationships appropriately, the perspectives of both parties involved need to be considered. Concerning the dynamics of attachment, change processes could be similar or different for the two parties in an attachment dyad. In this vein, the analysis of the stability and change of attachment in relationships presumes that both partners’ views must be assessed and analyzed jointly over time. Such an assessment of mutual attachments was used in studies on romantic couples (e.g., Seiffge-Krenke and Burk, 2012) and studies on mothers and their adolescent child (e.g., Cook and Kenny, 2005).

Although previous studies revealed important insights into the stability and change of attachment they are limited by the fact that attachment has been analyzed on the level of observed variables (e.g., Furmann et al., 2002; Cook and Kenny, 2005; Scharfe and Cole, 2006; Hiester et al., 2009; De Goede et al., 2012; Seiffge-Krenke and Burk, 2012) or manifest categories (e.g., Kirkpatrick and Hazan, 1994; Baldwin and Fehr, 1995; Davila et al., 1997; Lopez and Gormley, 2002; Pinquart et al., 2013). However, in the presence of measurement error, estimates of instability are confounded with unreliability. A low retest correlation coefficient, for example, could indicate high stability but low reliability, low stability and high reliability, or low stability and low reliability. To separate unreliability from instability, latent variable models are necessary (e.g., Little, 2013; Eid and Kutscher, 2014; McArdle and Nesselroade, 2014).

### The Stability of Working Models of Attachment

The stability of attachment is highly influenced by the stability of the working models of attachment. These working models contain the stored knowledge of interactions with an attachment figure and allow predicting future interactions and the optimal amount of proximity or avoidance (Bowlby, 1973; Mikulincer and Shaver, 2016). Fraley (2002) distinguished two perspectives on the mechanisms underlying stability and change of attachment. First, according to the prototype perspective, the attachment representations form a prototype that continues to shape attachment patterns throughout the life span (Fraley, 2002). Although the working models of attachment can change through new experiences, the underlying prototype remains stable (Fraley et al., 2011). Second, according to the revisionist perspective, new experiences update the working models of attachment and they can completely wipe out the initial working model of attachment over time (Fraley, 2002).

The two perspectives on stability and change of attachment lead to different expectations for the patterns of stability in attachment ratings (Fraley, 2002; Fraley et al., 2011). Under the revisionist perspective, stability (in this case defined as the correlation between two measurement occasions) should continuously decrease over longer test-retest intervals. Under the prototype perspective, stability might also decrease with longer test-retest intervals but should stabilize at a value greater than zero. That is, according to the prototype perspective, stability should not be affected by the distance between two measurement occasions once a certain distance is exceeded. The difference between the two perspectives can only be observed with more than two measurement occasions (Fraley et al., 2011). Previous studies contrasting the revisionist and the prototype perspective supported the prototype perspective with young adults (Fraley et al., 2011) and adolescents (Jones et al., 2018). However, in these studies stability and change were analyzed on the level of observed variables.

For such analyses, one approach is particularly useful. The revised version of the latent state-trait (LST-R) theory (Steyer et al., 2015) allows separating measurement error from stable trait components (e.g., prototype factors) and occasion-specific components in longitudinal measurements. The reported attachment of a person at one measurement occasion is seen as their current state score. This state score can be decomposed into a trait score and an occasion-specific score. The trait is defined as the expected score of the state variable for a specific person and time point, irrespective of the situational factors realized for the respective person at this time point (Steyer et al., 2015). Note that according to LST-R theory (Steyer et al., 2015), this trait is not necessarily stable over time, as persons may change across time. The occasion-specific score captures the deviation from this trait score at a measurement occasion. A person might rate their parental attachment below the actual trait score if she or he is in a bad mood or had an argument with their parent shortly before the measurement occasion. The rating might be above the trait score if the person is in a good mood or had a pleasant event with the parents. A working model (and especially a prototypical element of a working model) influences the expected state score and therefore poses an aspect of the trait. A stronger influence of a prototype in the working models of attachment should lead not only to higher correlations between the trait scores of different measurement occasions but also to a similar (above zero) correlation of one trait score with all later trait scores beyond a specific test-retest interval. Moreover, LST models were extended to LST models with autoregressive effects (Cole et al., 2005; Eid et al., 2017) that allow to represent both perspectives (revisionist and prototype perspective) and to analyze which perspective might be more appropriate.

### Aims of the Present Study

As outlined above, research on stability and change of parental attachment can profit from a relationship-specific view of attachment assessed by both emerging adults and their parents over time. In order to consider measurement error, latent variable models are needed that can separate measurement error from true instability. The main aim of the present study is to show how longitudinal latent variable models that have been developed in the context of multimethod research (Eid, 2000; Geiser, 2008; Koch, 2013) can be adapted to analyze the stability and change of attachment to parents over time. The new multi-rater LST model with autoregressive effects (MR-LST-AR model) presented in this article allows to quantify the degree of consistency between the parents and the emerging adults concerning the stability and change of their mutual attachments.

The secondary aim is to use these models to contribute to a more comprehensive understanding of stability and change of parental attachment in emerging adulthood, a critical transition period between adolescence and adulthood. In particular, we are interested in the following research questions:

(1) How stable is attachment of emerging adults to their parents and attachment of parents to their emerging adult children?

Based on previous findings, we expected a medium to high stability of the emerging adults’ attachment to their parents. We expected that prototypical elements in the attachment would influence the stability. Using the LST-models, we wanted to estimate the extent of the prototypical influence. Since there were no studies about the attachment of parents to their emerging adult children, we had no expectations as to whether their attachment would be more stable than the attachment of the emerging adults to their parents.

(2) How consistent is attachment within dyads?

For dyads of parents and their emerging adult children, we expected a high consistency for attachment. Consistency in this context refers to the mutual agreement in the attachments of emerging adults to their parents and their parents’ attachments to their emerging adult child, as quantified in correlational terms. We assume that a higher attachment of one member of the dyad strengthens the attachment of the other member such that attachments mutually stabilize each other. Therefore, we expected a higher consistency in the stable parts of attachment and a lower consistency in the short-term deviations.

## Methods

### Procedure and Design

Our investigation is part of a longitudinal study on the stability and change of attachment patterns of German emerging adults during their 1st year after high school graduation. The emerging adults in this study graduated from high school in 2014 and were recruited through presentations in schools before their graduation (a minority was recruited through other methods such as flyers, university fairs, and Facebook). Only emerging adults who graduated in 2014 in Germany were included in the study (besides that, there were no further criteria of inclusion or exclusion). The emerging adults who participated in the study are called *targets* in the following to distinguish them from the participating parents.

The data collection comprised four measurement occasions from September 2014 to June 2015 (with 3 months intervals between the measurement occasions). The targets were invited to an online questionnaire on each measurement occasion. On the first measurement occasion, each target also named one parent who was invited to an online questionnaire on each of the four measurement occasions. The targets were paid 12.50 Euro for each measurement occasion and an extra 50 Euro if they participated on all four measurement occasions. Parents took part in a lottery that awarded tablet computers and cinema tickets after the last measurement occasion. Data from this study were also used to explore issues related to self-perceptions and informant perceptions of loneliness (Luhmann et al., 2016), the relationship between attachment and well-being (Bohn et al., 2020), and methodological extensions of models (Holtmann et al., 2017, 2020; Koch et al., 2018).

### Sample

A total of *N* = 575 targets (379 female, *M*_*age*_ = 18.2, *SD*_*age*_ = 0.6) participated on at least one measurement occasion (T1: *N* = 558, T2: *N* = 463, T3: *N* = 428, T4: *N* = 429). A total of *N* = 462 parents (368 mothers and 94 fathers, *M*_*age*_ = 47.7, *SD*_*age*_ = 4.9) participated on at least one measurement occasion (T1: *N* = 405, T2: *N* = 384, T3: *N* = 341, T4: *N* = 323). For a more detailed information on the sample and the longitudinal study see Bohn et al. (2020).

### Scales and Measurement

Attachment was measured with the Relationship-Specific Attachment Scales (Asendorpf et al., 1997). The targets rated their attachment to the parent who they nominated to participate in the study. Parents rated their attachment to the target. The wording of the items was adapted to match the specific relationship. All responses were provided on a 5-point rating scale ranging from 1 (*strongly disagree*) to 5 (*strongly agree*).

The six items for attachment were merged into three parcels for the analysis. Each parcel comprised a positively worded item and an inversed negative item on the same aspect of attachment. The first parcel contained items on acceptance, the second parcel contained items on dependability, and the third parcel contained items on closeness. Higher values in all parcels reflected higher security (vs. insecurity) of attachment.

### Latent State-Trait Model With Autoregressive Effects

**FIGURE 1:**
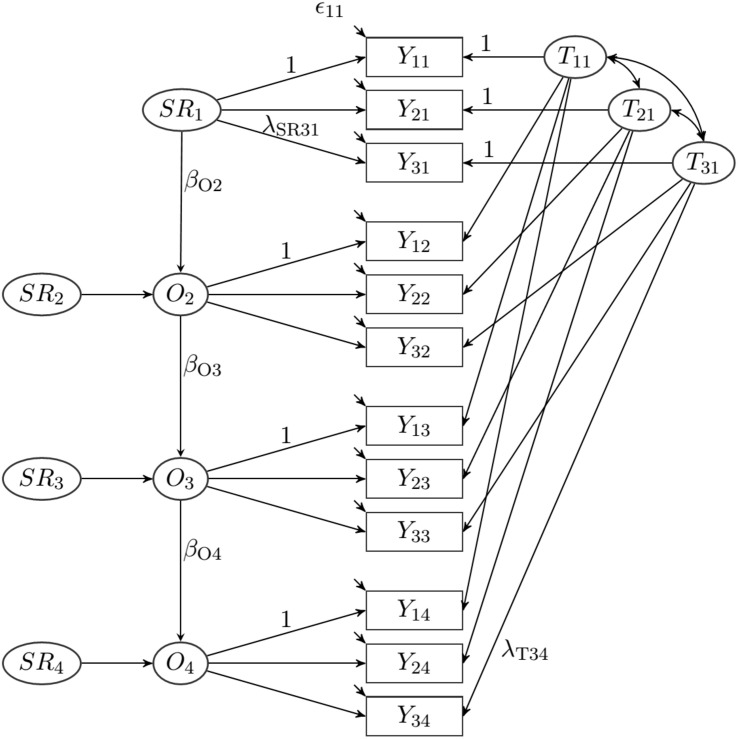
Latent state-trait model with autoregressive effects.

The analytic model was based on LST-R theory (Steyer et al., 2015). LST-R models allow disentangling trait components from occasion-specific components and measurement error in longitudinal measurements. To account for temporal dependency between adjacent measurement occasions, we used the extended LST model with autoregressive effects (LST-AR model, see Eid et al., 2012, 2017). An LST-AR model for three observed variables and four measurement occasions is depicted in Figure 1. LST-AR models have already been used in other studies to separate trait, occasion-specific, and accumulated situational effects (Scarpato et al., 2021).

This model is the starting point of our modeling approach. Eid et al. (2017) describe this model in detail and we will only sketch its major propositions concerning our application. In our application, the indicators *Y*_*il*_ (*i*: indicator; *l*: measurement occasion) are the observed attachment ratings of the targets on the four measurement occasions. The indicators represent different aspects of attachment (*i* = 1: acceptance; *i* = 2: dependability; *i* = 3: closeness). To describe the model, we start with the first indicator (*i* = 1) of the target’s rating on the first measurement occasion (*l* = 1). The observed variable *Y*_11_ is decomposed into a latent state variable *S*_11_ and a measurement error term *E*_11_:

(1) $$Y_{11}=a_{11}+S_{11}+E_{11}$$

where *a*_*11*_ is an intercept parameter. The state variable *S*_*11*_ represents latent individual differences in attachment on the first measurement occasion, corrected for measurement error *E*_*11*_. Hence, a high value on the variable *S*_*11*_ corresponds to a high self-reported true attachment of the target to the parent on the first measurement occasion. Because we centered the latent state variable, that means that *E*(*S*_11_) = 0, *a*_*11*_ equals the expected value of the observed variable *Y*_*11*_: *a*_11_=*E*(*S*_11_) The latent state variable *S*_*11*_ is then further decomposed into different components. The trait factor *T*_11_ captures the expected acceptance (as the first indicator of attachment) of the target on the first measurement occasion across all possible situations that could occur on the first measurement occasion. This model has indicator-specific trait factors. According to LST-R theory, the trait is defined as the expectation of the state variable for a specific person and time point, irrespective of the situational factors realized for the respective person at this time point (Steyer et al., 2015). In addition to the trait component, the latent state of a target at occasion 1 contains an occasion-specific component, which captures situational influences the target encounters or target-situation interactions at this specific measurement occasion. The state residual variable *SR*_1_ represents this occasion-specific deviation from the trait. A positive value on the factor *SR*_1_ indicates that the targets reported state attachment is higher than the target’s trait value. A higher attachment means a more secure attachment. Hence, the variables *S*_11_ and *Y*_11_ are decomposed in the following way:

(2) $$S_{11}=T_{11}+S\,R_{1}$$

(3) $$Y_{11}=a_{11}+T_{11}+S\,R_{1}+E_{11}$$

Because the state variable *S*_*11*_ was centered also the latent trait variable had to be centered (*E*(*T*_11_) = 0). Moreover, the expected values of *SR*_1_ and *E*_*11*_ are 0, as they are defined as residual variables (see Eid et al., 2017). The second indicator *Y*_*21*_ and the third indicator *Y*_*31*_ are decomposed similarly. Furthermore, it is assumed that state residual variables are unidimensional across indicators. This results in the following equations for the first measurement occasion (see Figure 1):

(4) $$Y_{21}=a_{21}+T_{21}+\lambda_{\text{SR}\,21}\,S\,R_{1}+E_{21}$$

(5) $$Y_{31}=a_{31}+T_{31}+\lambda_{\text{SR}\,31}\,S\,R_{1}+E_{31}$$

where λ_SR*il*_ are loading parameters.

The decomposition for measurement occasions *l* > 1 is slightly different. According to the LST-AR model, the later states are influenced by the trait of the first occasion and accumulated situational influences that occurred between the first and the current measurement occasion (Eid et al., 2017). Therefore, the latent state variables on measurement occasions *l* > 1 are composed of the weighted trait-factor *T*_*i1*_ and an occasion-specific factor *O*_*l*_, that is:

(6) $$S_{i\,l}=\lambda_{\text{T}\,i\,l}\,T_{i\,1}+\lambda_{\text{O}\,i\,l}\,{\text{O}}_{l}$$

and consequently:

(7) $$Y_{i\,l}=a_{i\,l}+\lambda_{\text{T}\,i\,l}\,T_{i\,1}+\lambda_{\text{O}\,i\,l}\,O_{l}+E_{i\,l}$$

where λ_T*i*1_ and λ_O*il*_ are loading parameters, with λ_O1*l*_ (*i* = 1) set to 1 for all occasions *l* for identification reasons. Moreover, the occasion-specific factor *O_l* is decomposed in the following way:

(8) $$O_{l}=\beta_{\text{O}\,l}\,O_{l-1}+S\,R_{l}\;\text{for all}\,l>1$$

(9) $$O_{1}=S\,R_{1}$$

where larger values of the autoregressive parameter β_O*l*_ represent a stronger influence of occasion-specific deviations of the last measurement occasion on the present measurement occasion. That is, the latent state variable on a measurement occasion *l* > 1 is a linear combination of the trait-factor *T*_*i1*_ and the occasion-specific factor *O*_*il*_. Furthermore, this occasion-specific factor is a linear combination of the occasion-specific factor of the previous measurement occasion and a state residual variable *SR*_*l*_ (Eid et al., 2017). That state residual variable *SR*_*l*_ (*l* > 1) captures those parts in the variable *Y*_*il*_ that are due to the situation or person-situation interactions and cannot be explained by the trait or carry-over effects from previous measurement occasions. Assuming measurement invariance over time in this model, the factor loadings of the occasion-specific variables *O_l* are set equal over time. Additionally, the variances of state residual variables *SR*_*l*_ and the autoregressive parameters β_*Ol*_ can be set equal over time to incorporate the assumption of a homogeneous change process.

Note that the model includes indicator-specific trait factors *T*_11_, *T*_21_, and *T*_31_ to account for potential indicator-specific effects. Assuming that all indicators measure attachment, the trait factors should strongly correlate, while a low correlation would be a sign for heterogeneity between the indicators.

#### Variance Decomposition

The variance of the indicators *Y*_*i1*_ of the first measurement occasion (*l* = 1) can be decomposed in the following way:

(10) $$\text{Var}\,\left(Y_{i\,1}\right)=\text{Var}\,\left(T_{i\,1}\right)+\lambda_{\text{SR}\,i\,l}^{2}\,\text{Var}\,\left(S\,R_{1}\right)+\text{Var}\,\left(E_{i\,l}\right)$$

For the later measurement occasions (*l* > 1), the variance of the indicators can be decomposed as follows:

(11) $$\text{Var}\,\left(Y_{i\,l}\right)=\lambda_{\text{T}\,i\,l}^{2}\,\text{Var}\,\left(T_{i\,1}\right)+\lambda_{\text{O}\,i\,l}^{2}\,\text{Var}\,\left(O_{l}\right)+\text{Var}\,\left(E_{i\,l}\right)$$

with,

(12) $$\text{Var}\,\left(O_{l}\right)=\beta_{\text{O}\,l}^{2}\,\text{Var}\,\left(O_{l-1}\right)+\text{Var}\,\left(S\,R_{l}\right)$$

Based on these variance decompositions, several coefficients that quantify relative variance proportions can be calculated. The reliability coefficient *Rel*(*Y*_*il*_) represents the proportion of an indicator’s variance that is true (error-free) variance and is given by:

(13) $$\text{Rel}\,\left(Y_{i\,l}\right)=1-\frac{\text{Var}\,\left(E_{i\,l}\right)}{\text{Var}\,\left(Y_{i\,l}\right)}$$

Other coefficients illustrate different aspects of stability and change of attachment. The time consistency coefficient *TCon*(*Y*_*il*_) can be calculated for the second and later measurement occasions (*l* > 1). The time consistency coefficient describes the proportion of an indicator’s true (error-free) variance that can be explained by observations on the same indicator on former measurement occasions. The time consistency coefficient *TCon*(*Y*_*il*_) is defined as:

(14) $$\text{TCon}\,\left(Y_{i\,l}\right)=\frac{\lambda_{\text{T}\,i\,l}^{2}\,\text{Var}\,\left(T_{i\,1}\right)+\lambda_{\text{O}\,i\,l}^{2}\,\beta_{\text{O}\,l}^{2}\,\text{Var}\,\left(O_{l-1}\right)}{\text{Var}\,\left(Y_{i\,l}\right)-\text{Var}\,\left(E_{i\,l}\right)}$$

The time consistency coefficient can be decomposed into two parts to separate the influence of the trait on the first measurement occasion from accumulated situational effects. The predictability by trait_1_ coefficient *Pred*_*trait1*_(*Y*_*il*_) describes the proportion of an indicator’s true (error-free) variance that can be predicted by the trait variable on the first measurement occasion. A high *Pred*_*trait1*_(*Y*_*il*_) indicates that the trait on the first measurement occasion is a good predictor for this measurement occasion. The predictability by trait_1_ coefficient *Pred*_*trait1*_(*Y*_*il*_) is defined as:

(15) $${\text{Pred}}_{\mathrm{trait1}}\,\left(Y_{i\,l}\right)=\frac{\lambda_{T\,i\,l}^{2}\,\text{Var}\,\left(T_{i\,1}\right)}{\text{Var}\,\left(Y_{i\,l}\right)-\text{Var}\,\left(E_{i\,l}\right)}$$

The unpredictability by trait_1_ coefficient *UPred*_*trait1*_(*Y*_*il*_) quantifies the time-consistent proportion of an indicator’s true variance that cannot be explained by the trait value on the first measurement occasion. This coefficient represents the influence of accumulated situational effects throughout the study. It can only be calculated after the first measurement occasion. The *UPred*_*trait1*_(*Y*_*il*_) is defined as:

(16) $${\text{UPred}}_{\mathrm{trait1}}\,\left(Y_{i\,l}\right)=\frac{\lambda_{\text{O}\,i\,l}^{2}\,\beta_{\text{O}\,l}^{2}\,\text{Var}\,\left(O_{l-1}\right)}{\text{Var}\,\left(Y_{i\,l}\right)-\text{Var}\,\left(E_{i\,l}\right)}$$

The predictability by trait_1_ coefficient and the unpredictability by trait_1_ coefficient add up to the time consistency coefficient.

The occasion-specificity coefficient *OS*(*Y*_*il*_) describes the proportion of an indicator’s true (error-free) variance that can be explained by the state residual variable. This variance is specific to a measurement occasion and cannot be explained by the trait factor of the first measurement occasion or carry-over effects from previous occasions. A high *OS* shows that the indicator is subject to strong occasional variations, i.e., a high proportion of the variables’ variance is due to unexplained occasion-specific situational and person-situation interaction effects. The occasion-specificity coefficient *OS*(*Y*_*il*_) is defined as:

(17) $$\text{OS}\,\left(Y_{i\,l}\right)=\frac{\lambda_{\text{O}\,i\,l}^{2}\,\text{Var}\,\left(S\,R_{l}\right)}{\text{Var}\,\left(Y_{i\,l}\right)-\text{Var}\,\left(E_{i\,l}\right)}$$

A potential prototype in the working model would be a part of the trait at the first measurement occasion. The prototype is supposed to be stable. So, the trait on the first measurement occasion can be used to predict the prototypical elements of the attachment at later measurement occasions and there should be no autoregressive effects. Therefore, the predictability by trait_1_ coefficient should approach a non-zero value for later measurement occasions under the prototype perspective. Under the revisionist perspective, the predictability by trait_1_ coefficient should get smaller with every measurement occasion and approach zero (see also Fraley, 2002) (the period covered in the present study, however, might be too short for the predictability by trait_1_ coefficient to actually reach zero).

In earlier studies that investigated these different perspectives (e.g., Fraley, 2002; Fraley et al., 2011), the correlations of the first measurement occasion with several later occasions were examined. To enhance comparability of our results with results from previous studies, we also calculate a measurement error-free correlation between the states on the later measurement occasions and the state on the first measurement occasion. For the second measurement occasion, this correlation is:

(18) $$r\,\left(S_{i\,1},S_{i\,2}\right)=\frac{\lambda_{T\,i\,2}\,\text{Var}\,\left(T_{i\,1}\right)+\lambda_{\text{SR}\,i\,1}\,\lambda_{\text{O}\,i\,2}\,\beta_{\text{O}\,2}\,\text{Var}\,\left(S\,R_{1}\right)}{\sqrt{\text{Var}\,\left(Y_{i\,1}\right)-\text{Var}\,\left(E_{i\,1}\right)}\,\sqrt{\text{Var}\,\left(Y_{i\,2}\right)-\text{Var}\,\left(E_{i\,2}\right)}}$$

For the third measurement occasion, the correlation is:

(19) $$r\,\left(S_{i\,1},S_{i\,3}\right)=\frac{\lambda_{T\,i\,3}\,\text{Var}\,\left(T_{i\,1}\right)+\lambda_{\text{SR}\,i\,1}\,\lambda_{\text{O}\,i\,3}\,\beta_{\text{O}\,2}\,\beta_{\text{O}\,3}\,\text{Var}\,\left(S\,R_{1}\right)}{\sqrt{\text{Var}\,\left(Y_{i\,1}\right)-\text{Var}\,\left(E_{i\,1}\right)}\,\sqrt{\text{Var}\,\left(Y_{i\,3}\right)-\text{Var}\,\left(E_{i\,3}\right)}}$$

For the fourth measurement occasion, the correlation is:

(20) $$r\,\left(S_{i\,1},S_{i\,4}\right)=\frac{\lambda_{T\,i\,4}\,\text{Var}\,\left(T_{i\,1}\right)+\lambda_{\text{SR}\,i\,1}\,\lambda_{\text{O}\,i\,4}\,\beta_{\text{O}\,2}\,\beta_{\text{O}\,3}\,\beta_{\text{O}\,4}\,\text{Var}\,\left(S\,R_{1}\right)}{\sqrt{\text{Var}\,\left(Y_{i\,1}\right)-\text{Var}\,\left(E_{i\,1}\right)}\,\sqrt{\text{Var}\,\left(Y_{i\,4}\right)-\text{Var}\,\left(E_{i\,4}\right)}}$$

The LST-AR model was used to examine the different aspects of stability and change of the targets’ attachment to their parents. The same model was also used to examine the parents’ attachment to their emerging adult children.

### Multi-Rater Latent State-Trait Model With Autoregressive Effects

**FIGURE 2:**
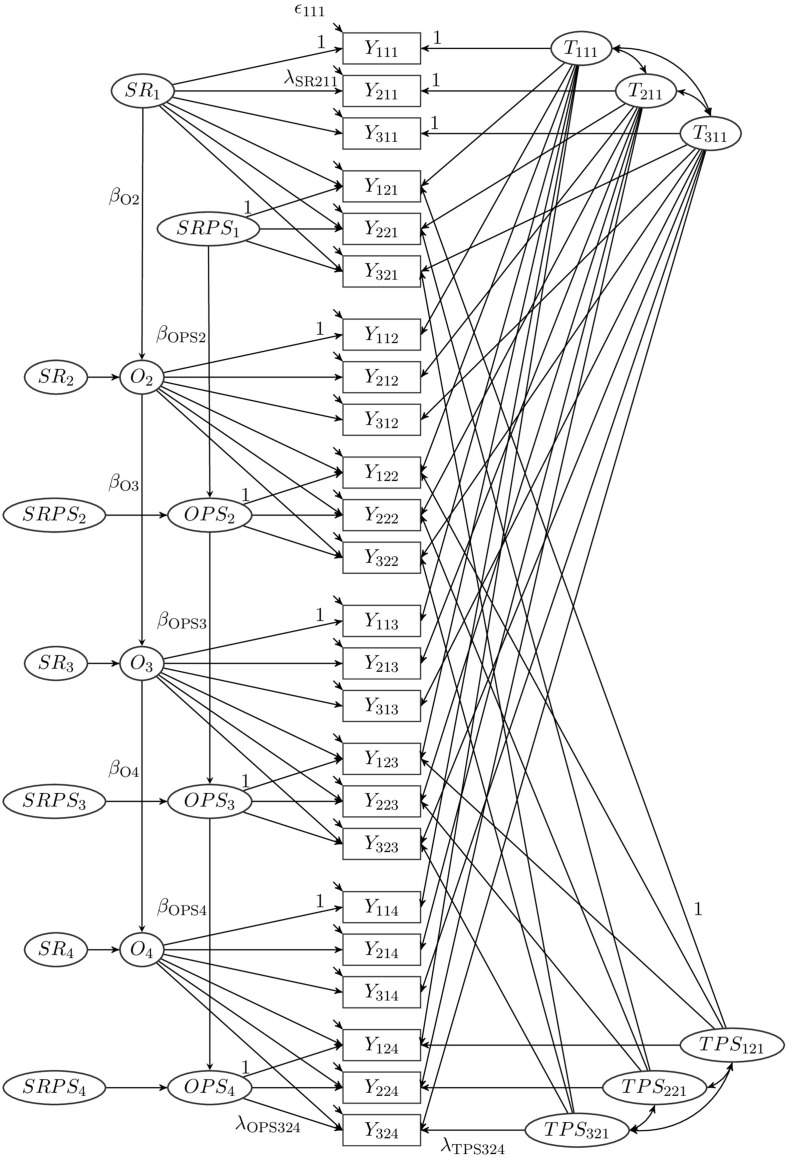
Multi-rater latent state-trait model with autoregressive effects.

To examine the convergence of the attachment ratings over time, we combined the ratings of the mutual attachments of parents and targets in one model. Our model is an extension of a model presented by Courvoisier et al. (2008), which combines the ideas of multitrait-multimethod analysis (MTMM; Campbell and Fiske, 1959) and autoregressive LST models (e.g., Cole et al., 2005; Eid et al., 2017). Confirmatory factor analysis MTMM models allow separating trait, method, and error components of a measurement. The specific MTMM model used for the present analysis is the correlated trait-correlated methods minus one (CT-C[M-1]) model (Eid, 2000; Eid et al., 2003), which is an appropriate model for analyzing MTMM data with structurally different methods (Eid et al., 2008, 2016; Nussbeck et al., 2009; Koch et al., 2018). In this model, a reference method is chosen (here: target report) and all other methods (here: parent report) are contrasted against this reference method (Eid, 2000; Eid et al., 2003). In our model, the target report and the parent report do not represent different methods in a narrow sense, but they represent the view of different raters on their mutual attachment. Figure 2 depicts the employed multi-rater LST-model with autoregressive effects (MR-LST-AR model) for attachment, measured by three indicators with two methods (target and parent report) at 4 measurement occasions.

The indicators *Y*_*ikl*_ (*i*: indicator; *k*: rater; *l*: measurement occasion) are the observed attachment ratings on the four measurement occasions. For the indicators of the targets’ ratings (*k* = 1), the decomposition of the variables is equivalent to the LST-AR model presented in Figure 1 and defined in Eqs. (2-9). The trait factors *T*_*i*__11_ and the occasion-specific factors *O*_*l*_ have the same meaning for the targets’ ratings as in the LST model. The decomposition of indicators of the parents’ ratings (*k* = 2) is explained in the following. In line with the CT-C(M-1) approach to multimethod modeling, we use the targets’ attachment to predict the parents’ attachment. This regression is included on the trait level (regressing the parents’ trait attachment on the targets’ trait attachment) as well as on the occasion-specific level (regressing the parents’ occasion-specific attachment on the targets’ occasion-specific attachment). The size of the respective standardized regression coefficients reflect the strength of the association between the targets’ and their parents’ attachment. These coefficients correspond to the standardized factor loadings in the model.

The component of the parents’ attachment that cannot be explained by the targets’ attachment is captured in the parent-specific trait factors *TPS*_*i*__2_*_1_* and the parent-specific occasion factors *OPS*_*l*_. Note that the parent-specific trait factors *TPS*_*i*__2_*_1_* are indicator-specific factors. As they are defined as residual factors, the factors *TPS*_*i*__2_*_1_* have a mean of zero and are uncorrelated with the respective trait factor *T*_*i*__1_*_1_* by definition. They represent the deviation of the parents’ trait attachment as measured by indicator *i* from the value expected based on the target’s trait attachment on the first measurement occasion. A positive value on the factor *TPS*_*i21*_ indicates that the parent’s trait attachment on the first measurement occasion is higher (meaning more secure) than expected based on the target’s trait attachment. Mean differences between the targets’ and parents’ attachments are captured in the intercepts of the corresponding observed variables since all trait factors are modeled with a mean of zero.

The occasion- and parent-specific factors *OPS*_*l*_ capture those parts of the variance in the parent ratings that are occasion-specific and not shared with the targets’ occasion-specific attachments. As the factors *OPS*_*l*_ are defined as residual factors with respect to the targets’ occasion-specific factors, they have a mean of zero and are uncorrelated with the respective occasion-specific factor *O*_*l*_ by definition. A positive value on *OPS*_*l*_ indicates that the parent’s momentary, occasion-specific attachment is higher than expected based on the target’s momentary, occasion-specific attachment.

Altogether the observed variables of the parent reports (*k* = 2) can be decomposed in the following way:

(21) $$Y_{i\,2\,l}=a_{i\,2\,l}+\lambda_{\text{T}\,i\,2\,l}\,T_{i\,11}+\lambda_{\text{TPS}\,i\,2\,l}\,T\,P\,S_{i\,21}+\lambda_{\text{O}\,i\,2\,l}\,O_{l}+\lambda_{\text{OPS}\,i\,2\,l}\,O\,P\,S_{l}+E_{i\,2\,l}$$

where the loading parameter λ_TPS*i*21_for the first measurement occasion (*l* = 1) is set to 1 for identification reasons, such that *TPS*_*i*__21_ is defined as the parent-specific trait factor at the first measurement occasion. Carry-over effects in the occasion- and parent-specific view are captured by the inclusion of autoregressive effects on the factors *OPS*_*l*_. The autoregressions can be expressed as:

(22) $$O\,P\,S_{l}=\beta_{\text{OPS}\,l}\,O\,P\,S_{l-1}+S\,R\,P\,S_{l}\;\text{for all}\,l>1$$

(23) $$O\,P\,S_{1}=S\,R\,P\,S_{1}$$

with *SRPS*_*l*_ being a state residual factor for the parent-specific attachment, that is, that part in the parents’ attachment that can neither be explained by the targets’ attachment on the same measurement occasion nor by previous deviations of the parents’ attachment from the targets’ attachment. Since all factors have a mean of zero, overall changes in the level of attachment are visible in changes of the intercepts *a*_*ikl*_.

#### Variance Components

The variance decomposition of the observed variables of the targets’ ratings (*k* = 1) is completely analogous to the LST-AR model presented above, compare Eqs. (10-12). The variance of the observed variables of the parents’ ratings (*k* = 2) can be decomposed in the following way:

(24) $$\text{Var}\,\left(Y_{i\,2\,l}\right)=\lambda_{\text{T}\,i\,2\,l}^{2}\,\text{Var}\,\left(T_{i\,11}\right)+\lambda_{\text{TPS}\,i\,2\,l}^{2}\,\text{Var}\,\left(T\,P\,S_{i\,21}\right)+\lambda_{\text{O}\,i\,2\,l}^{2}\,\text{Var}\,\left(O_{l}\right)+\lambda_{\text{OPS}\,i\,2\,l}^{2}\,\text{Var}\,\left(O\,P\,S_{l}\right)+\text{Var}\,\left(E_{i\,2\,l}\right)$$

with,

(25) $$\text{Var}\,\left(O\,P\,S_{1}\right)=\text{Var}\,\left(S\,R\,P\,S_{1}\right)$$

(26) $$\text{Var}\,\left(O\,P\,S_{l}\right)=\beta_{\text{OPS}\,l}^{2}\,\text{Var}\,\left(O\,P\,S_{l-1}\right)+\text{Var}\,\left(S\,R\,P\,S_{l}\right)\,\text{for all}\,l>1$$

Equations 11 and 12 also apply for the parents’ ratings (*k* = 2). Based on these variance decompositions, the same coefficients, as in the LST-AR model, can be calculated. These coefficients are displayed in the Supplementary Material and their meaning is the same as in the LST-AR model. However, the MR-LST-AR model allows to calculate additional coefficients that quantify different aspects of consistency between the raters (in our case, the targets and the parents). In this article, we use the term ‘rater consistency’ for the convergence between raters. This term should not be confused with the ‘time consistency’, which describes the consistency over time.

The variance of the parents’ ratings (*k* = 2) can be separated into a part that is explained by the targets’ ratings and a part that is specific to the parents. The rater specificity coefficient (*RS*(*Y*_*i*2*l*_)) refers to the proportion of true (error-free) variance in the parents’ ratings that is not shared with the targets’ ratings on a specific measurement occasion. The counterpart of the rater specificity is the rater consistency (*RCon*(*Y*_*i*2*l*_)) between the ratings of targets and the parents.

The rater specificity coefficient *RS*(*Y*_*i*2*l*_) is defined as:

(27) $$\text{RS}\,\left(Y_{i\,2\,l}\right)=\frac{\lambda_{\text{TPS}\,i\,2\,l}^{2}\,\text{Var}\,\left(T\,P\,S_{i\,21}\right)+\lambda_{\text{OPS}\,i\,2\,l}^{2}\,\text{Var}\,\left(O\,P\,S_{l}\right)}{\text{Var}\,\left(Y_{i\,2\,l}\right)-\text{Var}\,\left(E_{i\,2\,l}\right)}$$

The rater consistency coefficient *RCon*(*Y*_*i*2*l*_) is defined as:

(28) $$\text{RCon}\,\left(Y_{i\,2\,l}\right)=\frac{\lambda_{\text{T}\,i\,2\,l}^{2}\,\text{Var}\,\left(T_{i\,11}\right)+\lambda_{\text{O}\,i\,2\,l}^{2}\,\text{Var}\,\left(O_{l}\right)}{\text{Var}\,\left(Y_{i\,2\,l}\right)-\text{Var}\,\left(E_{i\,2\,l}\right)}$$

The *RS*(*Y*_*i*2*l*_) and the *RCon*(*Y*_*i*2*l*_) add up to 1. The square root of the consistency coefficient equals the true (error-free) correlation between the targets’ and the parents’ ratings. A high consistency coefficient (and therefore low rater specificity) indicates that those targets who are more securely attached to their parent (as compared to other targets) tend to have parents who are also more securely attached to them (as compared to other parents).

Analogously, coefficients of rater-specificity and a rater-consistency can be defined on the trait and the occasion-specific levels, as well as for time-consistent components and components predictable and unpredictable by trait_1_. To examine the rater consistency on the level of inter-individual trait differences that go back to the first time point, we can calculate the rater-consistent predictability by trait_1_ coefficient *RConPred*_*trait1*_(*Y*_*i*2*l*_). The *RConPred*_*trait1*_(*Y*_*i*2*l*_)captures that amount of variance in inter-individual differences that goes back to the trait on the first measurement occasion that is shared by targets and parents. The *RConPred*_*trait1*_(*Y*_*i*2*l*_) is defined as:

(29) $${\text{RConPred}}_{\mathrm{trait1}}\,\left(Y_{i\,2\,l}\right)=\frac{\lambda_{\text{T}\,i\,2\,l}^{2}\,\text{Var}\,\left(T_{i\,11}\right)}{\lambda_{\text{T}\,i\,2\,l}^{2}\,\text{Var}\,\left(T_{i\,11}\right)+\lambda_{\text{TPS}\,i\,2\,l}^{2}\,\text{Var}\,\left(T\,P\,S_{i\,21}\right)}$$

Its counterpart is the rater-specific predictability by trait_1_ coefficient *RSPred*_*trait1*_(*Y*_*i*2*l*_), which quantifies the degree to which variance due to inter-individual differences in trait attachment on the first measurement occasion is specific to the parents and not shared with the targets (see Supplementary Material for a mathematical definition). The two coefficients *RConPred*_*trait1*_(*Y*_*i*2*l*_) and *RSPred*_*trait1*_(*Y*_*i*2*l*_) add up to 1 for the same indicator. If the *RConPred*_*trait1*_(*Y*_*i*2*l*_) is high (and the *RSPred*_*trait1*_(*Y*_*i*2*l*_) therefore low) the influence of inter-individual differences in the trait values on the first measurement occasion on later occasions can be explained by attachment features that are shared by both targets and parents.

On the level of time-consistent components of attachment, the rater-consistent time consistency coefficient *RConTCon*(*Y*_*i2l*_) describes the proportion of the time consistent variance in the parents’ attachment that can be explained by the targets’ time consistent attachment. Time consistency in this context means that rater consistency is calculated with respect to the variance that carries over from former measurement occasions (by effects of trait_1_ or accumulated situational effects). The rater-consistent time consistency coefficient *RConTCon*(*Y*_*i2l*_) is defined as:

(30) $$\text{RConTCon}\,\left(Y_{i\,1\,l}\right)=\frac{\lambda_{\text{T}\,i\,2\,l}^{2}\,\text{Var}\,\left(T_{i\,11}\right)+}{\lambda_{\text{T}\,i\,2\,l}^{2}\,\text{Var}\,\left(T_{i\,11}\right)+\lambda_{\text{TPS}\,i\,2\,l}^{2}\,\text{Var}\,\left(T\,P\,S_{i\,21}\right)+}\frac{\lambda_{\text{O}\,i\,2\,l}^{2}\,\beta_{\text{O}\,l}^{2}\,\text{Var}\,\left(O_{l-1}\right)}{\lambda_{\text{O}\,i\,2\,l}^{2}\,\beta_{\text{O}\,l}^{2}\,\text{Var}\,\left(O_{l-1}\right)+\lambda_{\text{OPS}\,i\,2\,l}^{2}\,\beta_{\text{OPS}\,l}^{2}\,\text{Var}\,\left(O\,P\,S_{l-1}\right)}$$

The rater-consistent occasion specificity coefficient *RConOS*(*Y*_*i*2*l*_) describes the percentage of state-residual variance in the parents’ attachment ratings that is consistent with the state-residual variance in the targets’ ratings. This coefficient is defined as:

(31) $$\text{RConOS}\,\left(Y_{i\,2\,l}\right)=\frac{\lambda_{\text{O}\,i\,2\,l}^{2}\,\text{Var}\,\left(S\,R_{l}\right)}{\lambda_{\text{O}\,i\,2\,l}^{2}\,\text{Var}\,\left(S\,R_{l}\right)+\lambda_{\text{OPS}\,i\,2\,l}^{2}\,\text{Var}\,\left(S\,R\,P\,S_{l}\right)}$$

That is, the *RConOS*(*Y*_*i*2*l*_) captures the amount of unexplained variance in attachment at a specific measurement occasion that is shared between parents and targets. A high *RConOS*(*Y*_*i*2*l*_) indicates that unexpected temporal deviations in attachment values (from those attachment values expected based on previously observed attachment patterns) covary between targets and their parents. That is, a higher attachment of the target on a given time point (as compared to the attachment that is expected based on the targets’ previous attachment to the parent) is associated with a higher than expected attachment of the parent at this time point. The square root of the consistent occasion specificity coefficient can be interpreted as a measurement error-free correlation between the unexplained occasion-specific deviations of targets’ and parents’ attachments (these unexplained occasion-specific deviations are captured in the state residual variables). In applications with strong measurement invariance (and therefore equal variances and loadings of the occasion specific factors), the rater-consistent occasion specificity for the same indicator is equal for all measurement occasions.

The rater-specific occasion specificity coefficient can be calculated as a counterpart of the rater-consistent occasion specificity coefficient. Additionally, further coefficients (like the rater-consistent unpredictability by trait_1_ coefficient) can be specified that might be useful in other applications of the MR-LST-AR model. An overview of all possible coefficients can be found in the Supplementary Material.

### Estimation

To examine the stability and change of attachment as well as the convergence between targets’ and parents’ attachments, we applied the models introduced above to the item parcels capturing the three attachment dimensions acceptance, dependability, and closeness. In a first step, we fitted two separate LST-AR models, one for the targets and one for the parents. In a second step, we estimated the MR-LST-AR model.

All models were estimated with the robust maximum likelihood estimator in MPlus 8.0 (Muthén and Muthén, 1998-2017). Full information maximum likelihood was used to handle missing values. The confidence intervals for the variance components were estimated via bootstrapping (5000 samples) using the maximum likelihood estimator. We assumed measurement invariance with equal loadings of the occasion-specific factors and equal variances of the state residual factors over time. In addition, to examine gender effects, the LST-AR models were run again separately for male and female targets.

We used the χ^2^-test, the CFI, and the RMSEA to examine the goodness-of-fit. A non-significant χ^2^-test (or at least a value of χ^2^ < 2^∗^*df*), a CFI > 0.97 and a RMSEA < 0.05 are signs of a good model fit (Schermelleh-Engel et al., 2003).

## Results

### Model Fit Criteria

All models fit the data well (targets’ LST-AR model: χ^2^(47) = 60.218; *p* = 0.093; RMSEA = 0.022; CFI = 0.993; parents’ LST-AR model: χ^2^(47) = 68.399; *p* = 0.022; RMSEA = 0.031; CFI = 0.981; MR-LST-AR model: χ^2^(217) = 261.321; *p* = 0.021; RMSEA = 0.018; CFI = 0.989).

### LST-AR Model

The first research question on the stability and change of attachment can be answered with the LST-AR models.

Variance coefficients and intercepts of the LST-AR model of targets’ attachment are displayed in Table 1. The reliability of the indicators ranged from 0.660 to 0.803, which indicates good reliability.

**TABLE 1:** Results of the LST-AR model of targets’ attachment.

	**a**_**i***j*_	**Rel**	**OS**	**TCon**	**Pred**	**Unpred**	**r**(**S_i1_**,**S**_**i***l*_)
*Y*_*11*_	4.52	0.720 [0.63, 0.82]	0.381 [0.26, 0.52]	0.619 [0.48, 0.74]	0.619 [0.48, 0.74]		
*Y*_*21*_	4.48	0.666 [0.56, 0.78]	0.413 [0.29, 0.56]	0.587 [0.44, 0.71]	0.587 [0.44, 0.71]		
*Y*_*31*_	4.08	0.660 [0.59, 0.72]	0.180 [0.11, 0.28]	0.820 [0.72, 0.89]	0.820 [0.72, 0.89]		
*Y*_*12*_	4.49	0.793 [0.71, 0.87]	0.330 [0.22, 0.46]	0.670 [0.55, 0.78]	0.617 [0.45, 0.75]	0.053 [0.01, 0.15]	0.760 [0.65, 0.85]
*Y*_*22*_	4.46	0.669 [0.57, 0.76]	0.398 [0.27, 0.53]	0.602 [0.47, 0.73]	0.539 [0.33, 0.70]	0.063 [0.01, 0.20]	0.724 [0.62, 0.81]
*Y*_*32*_	4.02	0.789 [0.72, 0.85]	0.141 [0.08, 0.22]	0.859 [0.78, 0.92]	0.836 [0.71, 0.91]	0.022 [0.00, 0.09]	0.892 [0.84, 0.94]
*Y*_*13*_	4.42	0.773 [0.68, 0.87]	0.328 [0.22, 0.44]	0.672 [0.56, 0.78]	0.611 [0.38, 0.75]	0.060 [0.01, 0.23]	0.671 [0.56, 0.77]
*Y*_*23*_	4.43	0.787 [0.70, 0.87]	0.360 [0.24, 0.49]	0.640 [0.51, 0.76]	0.574 [0.32, 0.73]	0.066 [0.01, 0.26]	0.642 [0.53, 0.74]
*Y*_*33*_	3.95	0.799 [0.73, 0.86]	0.138 [0.08, 0.21]	0.862 [0.79, 0.92]	0.837 [0.69, 0.91]	0.025 [0.00, 0.12]	0.854 [0.78, 0.91]
*Y*_*14*_	4.42	0.716 [0.61, 0.82]	0.403 [0.27, 0.53]	0.597 [0.47, 0.73]	0.521 [0.27, 0.69]	0.076 [0.01, 0.30]	0.593 [0.46, 0.71]
*Y*_*24*_	4.41	0.712 [0.61, 0.82]	0.376 [0.24, 0.51]	0.624 [0.49, 0.76]	0.554 [0.29, 0.73]	0.071 [0.01, 0.30]	0.595 [0.46, 0.72]
*Y*_*34*_	4.00	0.803 [0.74, 0.87]	0.147 [0.09, 0.22]	0.853 [0.78, 0.91]	0.826 [0.66, 0.90]	0.028 [0.00, 0.14]	0.833 [0.75, 0.90]

*a_ij_, intercept; Rel, reliability coefficient; OS, occasion specificity coefficient; TCon, time consistency coefficient; Pred, predictability by trait_1_ coefficient; Unpred, unpredictability by trait_1_ coefficient; *r*(*S*_1_,*S*_*l*_), measurement error-free correlation between this measurement occasion and the first measurement occasion. *Y*_*ij*_ with *i*, indicator (1, acceptance; 2, dependability; 3, closeness); *l*, measurement occasion; the bootstrapped 95%-confidence intervals in parenthesis.*

The stability over time was generally high, as indicated by time consistency coefficients *TCon*(*Y*_*il*_) ranging from 0.597 to 0.672 for the acceptance indicators, from 0.602 to 0.640 for the dependability indicators, and from 0.853 to 0.862 for the closeness indicators. Time consistency coefficients of the same indicator were comparably high for all measurement occasions. Still, closeness had a higher time consistency than the other two indicators (with no overlap in the confidence intervals). The corresponding occasion specificity coefficients *OS*(*Y*_*il*_) ranged from 0.138 to 0.403.

The predictability by trait_1_ coefficient *Pred*_*trait1*_(*Y*_*il*_) ranged from 0.521 to 0.617 for the acceptance indicators, from 0.539 to 0.574 for the dependability indicators and from 0.826 to 0.837 for the closeness indicators. That is, the closeness trait value on the first measurement occasion could explain over 80% of the true variance of the closeness indicators on the second and later measurement occasions. The values of *Pred*_*trait1*_(*Y*_*il*_) were similar for the different measurement occasions. That is, time consistencies are largely due to a high impact of interindividual differences in the traits at the first measurement occasion. The unpredictability by trait_1_ coefficients *UPred*_*trait1*_(*Y*_*il*_) were smaller than 0.08 in all cases, indicating that accumulated situational effects can explain only 8% or less in true interindividual differences at later time points. Such accumulated situational effects are carry-over effects from occasion-specific interindividual differences at previous time points.

The correlations of the latent attachment states on later measurement occasions (*S*_*il*_) with the state on the first measurement occasion (*S*_*i1*_) decreased throughout the study (from 0.760 to 0.593 for acceptance and from 0.724 to 0.595 for dependability). For closeness, these correlations also decreased from 0.892 to 0.833, which was still a high test-retest correlation. The intercepts show that the targets rated their closeness slightly lower as compared to their acceptance and dependability, with no changes in the mean level of attachment over time.

In the model for the targets’ attachment, trait factor correlations between trait acceptance, dependability, and closeness at the first measurement occasions medium to high, with *r* = 0.506 for acceptance and dependability, *r* = 0.544 for acceptance and closeness, *r* = 0.668 for dependability and closeness. These trait correlations indicate that the three facets of attachment were strongly related, with every facet also capturing a unique part of attachment.

Variance coefficients and intercepts of the LST-AR model of parents’ attachment are displayed in Table 2. Reliability coefficients ranged from 0.582 to 0.745 for most indicators, indicating good reliability. For *Y*_*22*_ the reliability coefficient was only 0.498, which showed low reliability of the dependability indicator on the second measurement occasion.

**TABLE 2:** Results of the LST-AR model of parents’ attachment.

	**a**_**i***j*_	**Rel**	**OS**	**TCon**	**Pred**	**Unpred**	**r**(**S_i1_**,**S**_**i***l*_)
*Y*_*11*_	4.53	0.726 [0.61, 0.91]	0.225 [0.11, 0.35]	0.775 [0.65, 0.89]	0.775 [0.65, 0.89]		
*Y*_*21*_	4.42	0.584 [0.46, 0.72]	0.128 [0.07, 0.21]	0.872 [0.79, 0.93]	0.872 [0.79, 0.93]		
*Y*_*31*_	4.29	0.667 [0.53, 0.95]	0.241 [0.13, 0.37]	0.759 [0.63, 0.87]	0.759 [0.63, 0.87]		
*Y*_*12*_	4.55	0.697 [0.52, 0.85]	0.263 [0.16, 0.40]	0.737 [0.60, 0.84]	0.733 [0.46, 0.84]	0.004 [0.00, 0.20]	0.783 [0.64, 0.90]
*Y*_*22*_	4.52	0.498 [0.37, 0.64]	0.201 [0.10, 0.34]	0.799 [0.66, 0.90]	0.796 [0.53, 0.90]	0.003 [0.00, 0.17]	0.853 [0.76, 0.92]
*Y*_*32*_	4.32	0.680 [0.55, 0.81]	0.286 [0.14, 0.50]	0.714 [0.50, 0.86]	0.710 [0.24, 0.86]	0.004 [0.00, 0.29]	0.766 [0.66, 0.86]
*Y*_*13*_	4.57	0.597 [0.44, 0.77]	0.327 [0.20, 0.48]	0.673 [0.52, 0.80]	0.668 [0.33, 0.79]	0.005 [0.00, 0.33]	0.723 [0.59, 0.83]
*Y*_*23*_	4.52	0.582 [0.43, 0.72]	0.162 [0.08, 0.28]	0.838 [0.72, 0.92]	0.836 [0.54, 0.92]	0.002 [0.00, 0.22]	0.856 [0.76, 0.93]
*Y*_*33*_	4.29	0.745 [0.62, 0.86]	0.252 [0.12, 0.45]	0.748 [0.55, 0.88]	0.744 [0.23, 0.88]	0.004 [0.00, 0.37]	0.755 [0.58, 0.87]
*Y*_*14*_	4.60	0.666 [0.53, 0.82]	0.303 [0.18, 0.44]	0.697 [0.56, 0.82]	0.692 [0.35, 0.80]	0.004 [0.00, 0.36]	0.733 [0.59, 0.83]
*Y*_*24*_	4.55	0.642 [0.49, 0.80]	0.162 [0.08, 0.27]	0.838 [0.74, 0.92]	0.836 [0.57, 0.91]	0.002 [0.00, 0.23]	0.854 [0.75, 0.92]
*Y*_*34*_	4.34	0.706 [0.57, 0.86]	0.270 [0.13, 0.44]	0.730 [0.56, 0.87]	0.726 [0.20, 0.86]	0.004 [0.00, 0.44]	0.743 [0.51, 0.86]

*a_ij_, intercept; Rel, reliability coefficient; OS, occasion specificity coefficient; TCon, time consistency coefficient; Pred, predictability by trait_1_ coefficient; Unpred, unpredictability by trait_1_ coefficient; *r*(*S*_1_,*S*_*l*_), measurement error-free correlation between this measurement occasion and the first measurement occasion. *Y*_*ij*_ with *i*, indicator (1, acceptance; 2, dependability; 3, closeness); *l*, measurement occasion; the bootstrapped 95%-confidence intervals in parenthesis.*

The stability of the parent’s attachment was high. The time consistency coefficient *TCon*(*Y*_*il*_) ranged from 0.673 to 0.737 for the acceptance indicators, from 0.799 to 0.838 for the dependability indicators and from 0.714 to 0.748 for the closeness indicators. The differences between the different indicators were smaller than in the LST-AR model of the targets’ attachment. The corresponding occasion specificity coefficients *OS*(*Y*_*il*_) ranged from 0.162 to 0.327.

The predictability by trait_1_ coefficient *Pred*_*trait1*_(*Y*_*il*_) ranged from 0.668 to 0.733 for the acceptance indicators, from 0.796 to 0.836 for the dependability indicators and from 0.710 to 0.744 for the closeness indicators. This means that in all cases a large proportion of the true variance in the attachment ratings of the second and later measurement occasion could be explained by the trait value at the first measurement occasion. The values of *Pred*_*trait1*_(*Y*_*il*_) showed no systematic pattern over time. With values of 0.005 and below, the unpredictability by trait_1_ coefficient *UPred*_*trait1*_(*Y*_*il*_) showed that accumulated situational effects were practically non-existent.

The same was true for the correlations of the latent state values of later measurement occasions with the first measurement occasion. The measurement error-free correlations of the latent states of the second, third, and fourth measurement occasion with those of the first measurement occasion were similar with a huge overlap in the confidence intervals and no visible trend. The correlations for the acceptance and the closeness indicators were similar with values between 0.723 and 0.783; the correlations for dependability were higher with values between 0.853 and 0.856. The intercepts showed no indication of changes in the mean level of attachment over time.

In the LST-AR model of parents’ attachment, the correlation between trait acceptance and trait dependability was *r* = 0.565, between trait acceptance and trait closeness *r* = 0.700, and between trait dependability and trait closeness *r* = 0.539.

To examine gender differences, we calculated the LST-AR model for the female and male targets separately. Both models had an acceptable model fit and all results can be found in the Supplementary Material. The were no global differences between coefficients of the male sample and the coefficients of the female sample. For the dependability items, time consistency and predictability were slightly higher in the female sample. For acceptance, time consistency and predictability were slightly higher in the male sample. We also calculated two LST-AR models separately for mothers and fathers. While the model fit was good for the mother sample, the model fit was bad for the father sample. The model of the father sample had also theta estimation problems and therefore no trustworthy results. The estimation problems are likely due to the rather sample size of fathers that is too small and not appropriate for such complex structural equation models. Therefore, only the results of the mother sample are displayed in the Supplementary Material.

### MR-LST-AR Model

All coefficients calculated and reported for the LST-AR models are similar in the MR-LST-AR model with only minor deviations and are therefore not repeated here. The results for all coefficients concerning different aspects of rater consistency are displayed in Table 3 (more model parameters can be found in the Supplementary Material).

**TABLE 3:** Results of the MR-LST-AR model.

	**RS**	**RCon**	**$\sqrt{\text{R}\,\text{Con}}$**	**RConOS**	**RConTCon**	**RConPred**
*Y*_*121*_	0.835 [0.68, 0.93]	0.165 [0.07, 0.32]	0.406 [0.26, 0.56]	0.068 [0.01, 0.21]		
*Y*_*221*_	0.910 [0.74, 0.97]	0.090 [0.03, 0.26]	0.300 [0.16, 0.51]	0.067 [0.00, 0.27]		
*Y*_*321*_	0.805 [0.67, 0.91]	0.195 [0.09, 0.33]	0.442 [0.29, 0.57]	0.051 [0.00, 0.21]		
*Y*_*122*_	0.793 [0.61, 0.91]	0.207 [0.09, 0.39]	0.455 [0.30, 0.63]	0.068 [0.01, 0.21]	0.254 [0.09, 0.49]	0.251 [0.09, 0.51]
*Y*_*222*_	0.846 [0.65, 0.95]	0.154 [0.05, 0.35]	0.392 [0.23, 0.59]	0.067 [0.00, 0.27]	0.268 [0.09, 0.54]	0.266 [0.09, 0.55]
*Y*_*322*_	0.792 [0.67, 0.89]	0.208 [0.11, 0.33]	0.456 [0.36, 0.58]	0.051 [0.00, 0.21]	0.268 [0.14, 0.44]	0.266 [0.14, 0.58]
*Y*_*123*_	0.846 [0.65, 0.95]	0.154 [0.05, 0.35]	0.392 [0.22, 0.56]	0.068 [0.01, 0.21]	0.194 [0.05, 0.41]	0.189 [0.04, 0.43]
*Y*_*223*_	0.921 [0.60, 0.99]	0.079 [0.01, 0.40]	0.281 [0.10, 0.63]	0.067 [0.00, 0.27]	0.081 [0.00, 0.48]	0.079 [0.00, 0.48]
*Y*_*323*_	0.795 [0.67, 0.89]	0.205 [0.11, 0.33]	0.453 [0.32, 0.58]	0.051 [0.00, 0.21]	0.254 [0.12, 0.43]	0.252 [0.12, 0.57]
*Y*_*124*_	0.837 [0.66, 0.94]	0.163 [0.06, 0.34]	0.404 [0.25, 0.58]	0.068 [0.01, 0.21]	0.201 [0.06, 0.43]	0.197 [0.05, 0.45]
*Y*_*224*_	0.885 [0.71, 0.96]	0.115 [0.04, 0.29]	0.339 [0.19, 0.54]	0.067 [0.00, 0.27]	0.123 [0.03, 0.33]	0.121 [0.02, 0.34]
*Y*_*324*_	0.848 [0.74, 0.93]	0.152 [0.07, 0.26]	0.390 [0.27, 0.51]	0.051 [0.00, 0.21]	0.187 [0.08, 0.33]	0.185 [0.07, 0.45]

*RS, rater specificity coefficient; RCon, rater consistency coefficient; $\sqrt{\text{RCon}}$, measurement error free correlation between target and parent ratings; RConOS, rater-consistent occasion specificity coefficient; RConTCon, rater-consistent time consistency coefficient; RConPred, rater-consistent predictability by trait_1_ coefficient; *Y*_*ikl*_ with *i*, indicator (1, acceptance; 2, dependability; 3, closeness); *k*, rater (2, parent); *l*, occasion of measurement; the bootstrapped 95%-confidence intervals in parenthesis.*

The rater consistency coefficients *RCon*(*Y*_*i*2*l*_) ranged from 0.079 to 0.208. That is, 8 to 20% of the variance in true, measurement-error free inter-individual differences in the parents’ attachments can be explained by inter-individual differences in the targets’ attachments at a given measurement occasion. There were no systematic differences between the three attachment dimensions, with large overlaps in the confidence intervals. This corresponds to measurement error-free correlations between the targets’ and parents’ attachments ranging from 0.281 to 0.455, implying a medium to very large effect size (Funder and Ozer, 2019).

The rater-consistent predictability by trait_1_ coefficient *RConPred*_*trait1*_(*Y*_*i*2*l*_) ranged from 0.079 to 0.266, with no systematic differences between the indicators. This means that there was a meaningful overlap in those parts of the ratings of targets’ and parents’ that could be explained by the trait value at the first measurement occasion. The rater-consistent time consistency coefficients *RConTCon*(*Y*_*i*2*l*_) ranged from 0.081 to 0.268. Therefore, the time consistent parts in the attachments of parents and targets correlated between 0.285 and 0.518, implying a medium to very large effect (Funder and Ozer, 2019). This means that the stable and time consistent elements in the ratings of targets and parents were highly correlated.

The rater-consistent occasion specificity coefficient *RConOS*(*Y*_*i*2*l*_) ranged from 0.051 to 0.068. Because of measurement invariance settings (equal variances of the state residual factors and equal occasion-specific loadings over time), the rater-consistent occasion specificity coefficients were equal across measurement occasions. Only a small percentage of the state residual variance in parents’ attachments was shared with the targets. The corresponding measurement error-free correlations ranged from 0.226 to 0.261, implying a medium effect size (Funder and Ozer, 2019). There were no systematic differences between the indicators concerning the *RConOS*.

## Discussion

### Applicability of the MR-LST-AR Model

The MR-LST-AR model is a new model that combines the LST-AR model with the CTC(M-1) model (Eid, 2000; Eid et al., 2003, 2008; Koch, 2013). In the CTC(M-1) model, one method is chosen as a reference method and all other methods are contrasted against this reference method. In our case, the model is used for attachment ratings in dyads. Therefore, the consistency in this model is not a sign for agreement between methods, but for the agreement of both raters concerning the quality of their mutual attachments. By extending the scope of application of the CTC(M-1) model, the present study further illustrates that the CTC(M-1) model can address a wide range of psychological research questions.

In the model, the ratings of the target were chosen as a reference. Therefore, the attachment of the parents was regressed on the attachment of the target and the parent-specific factors captured the residuum. The decision to choose the targets’ rating as a reference was based on the substantial perspective of this study. The emerging adults were the starting point of this examination of attachment. It would also be possible to choose the parents as a reference and to set up the model accordingly. In that case, we could examine the deviation of the emerging adult’s attachment from the expected level based on the parent’s attachment. While the stability and the model fit would not be affected by the chosen reference, the consistency coefficient would differ.

### Stability of Attachment (LST-AR Models)

The second aim of the present study was to examine stability and change of mutual attachments between emerging adults and their parents. To do so, we used LST-R theory and introduced a new multi-rater latent state-trait model. The degree of attachment security versus insecurity was relatively stable for the targets as well as for the parents. Observed stability was primarily due to inter-individual differences in trait attachment at the first measurement occasion (measured by predictability by trait_1_). Accumulated situational effects were practically absent in the parents’ attachment, and they were small in the targets’ attachment. The low influence of accumulated situational effects is a sign of the low plasticity of attachment. The results indicate that this plasticity is even lower for the parents. This can be due to the higher age of the parents or because the parents had developed a more stable model of their attachment representations. At the same time, the targets experienced their 1st year after high school graduation. The changes in this year, like leaving the parental home or starting their courses at the university or elsewhere, might lead to new experiences that allow for some small adjustments in the targets’ attachment to their parents.

The equally high predictability by trait_1_ coefficients over the entire course of the study and the low autoregressive effects support the prototype perspective. The predictability by trait_1_ coefficients were decreasing only for acceptance in the model of the targets’ attachment. In all other cases, the trait values on the first measurement occasion were equally predictive for all later measurement occasions. Such a pattern is hard to explain under the revisionist perspective. Furthermore, the period of this study represents turbulent times in the 1st year after high school graduation. The emerging adults made many new experiences, but the predictability by trait_1_ coefficients of dependability and closeness were not affected by that. Overall, these results are more in line with the prototype perspective stating that there are elements of the working models of attachment that are not changed (at least not over 1 year) by situational influences.

While the correlations between the states of later measurement occasions and the first measurement occasion were stable for the parents, they were decreasing in the model of the targets’ attachment. These decreasing correlations are caused by accumulating situational effects. This means that there are changes in the traits’ working models of attachment. The prototype, however, is part of the trait (at every measurement occasion). The influence of the trait of the first measurement occasion is not decreasing for dependability and closeness. The distinction between trait and occasion-specific effects in LST-R models allows examining the prototype with greater precision.

The Relationship-Specific Attachment Scales has items on acceptance, dependability, and closeness. In this study, the three aspects of attachment showed some heterogeneity and different stability. While closeness was the most stable aspect in the model of the targets, this was not the case in the model of the parents. Although the 1st year after high school graduation is a year with many changes, the closeness of the targets was extremely stable on the mean level and in the inter-individual differences. Closeness is less connected to actual behavior than dependability and acceptance. Acceptance can be expressed or shown more easily than closeness. Dependability can be observed directly. Closeness needs to be felt, and new experiences might less influence this feeling.

### Consistency Between Parent and Target (MR-LST-AR Model)

The mutual attachments of the emerging adults and their parents showed medium to large correlations, indicating that emerging adults who are more securely attached to their parents tend to have parents who are more securely attached to them. However, the rater specificity coefficients around 0.80 and higher also show that more than three-quarters of the measurement error-free variance was specific to the parents. This means that the mutual attachments of parents and emerging adults cannot be seen as a characteristic of their relationship but as characteristics of each person within the dyadic relationship. Nevertheless, the correlation also indicates that the mutual attachments are not independent of each other.

The rater-consistent time consistency coefficient showed that around 20% of the time consistent variance of the parents’ attachment was shared with the targets’ time consistent attachment. The trait value (which would include a possible prototype) seems to be the most important factor behind the consistency. These coefficients can be transformed into correlations from 0.40 to 0.50 between the time consistent attachments of parents and emerging adults. These correlations were smaller for dependability at the third and fourth measurement occasion. A possible reason is that dependability can be more one-sided in a relationship than the other aspects of a relationship. It is possible that one person in a dyadic relationship (e.g., the emerging adult) relies on the second person (e.g., the parent) but not vice versa. Such a constellation seems less likely regarding closeness and acceptance.

The rater consistent occasion specificity coefficients were very similar for the different aspects of attachment. Around 6% of the occasion-specific variance of the parents’ ratings was shared with the targets’ rating. Therefore, the occasion-specific deviations of targets and parents had a correlation around 0.25. This correlation between the occasion-specific deviations was smaller than the correlation between the stable attachments. This means that the consistency between the mutual attachments of emerging adults and their parents is stronger in the long run than it is for short term changes. Nevertheless, this small to medium correlation shows that parents and emerging adults have at least some commonality in their updating of their mutual attachments.

### Therapeutic Implications

Psychotherapy can change attachment security (Mikulincer and Shaver, 2016). Psychotherapy can be considered a special interpersonal experience. Our study shows that emerging adults’ attachment changes through cumulative experiences. Therapy might make use of this mechanism. The high correlations between the mutual attachments of emerging adults and parents show that it may be useful to involve both parties in therapy to change attachment. The higher stability in the parents’ attachments may mean that therapeutic attachment change is more difficult for them.

### Limitations

Our study has some important limitations. First, it is important to note that the targets choose the participating parent. For targets who have different attachments to their parents (maybe because the relationship to one parent is ruined after a complicated divorce), they should have invited the parent they have the closest relationship with. Targets who have a functional relationship with both parents used different criteria to choose the parent. We asked the remaining targets after the last measurement occasion, and the answers ranged from a better attachment over more free time of this parent to technical reasons (e.g., only one parent uses the internet). It is reasonable to assume that the reported attachment to the chosen parent was better than or equal to the attachment to the not chosen parent, but we cannot be sure about this.

Second, our study did not investigate the possible mechanisms behind the stability and change of attachment directly. The prototype perspective and the revisionist perspective lead to different expectations regarding the shape of repeated test-retest correlations (Fraley et al., 2011). This is an indirect way to compare the two perspectives, and our results can only be indirect support for the prototype perspective.

Third, our study had a limited period of 9 months between the first and last measurement occasion. Many change processes in the development of attachment likely need longer studies to be observed. The time spans between the four measurement occasions were fixed, so we have no information about the attachment between the measurement occasions.

Fourth, the 1st year after high school graduation is very volatile and the results cannot be generalized to other life periods. However, it is interesting to see that attachment is so stable in a time of so many changes in life.

## Conclusion

The LST-AR models were able to show the high degree of stability of the attachments between emerging adults and their parents. The results foster the prototype perspective of a stable working model of attachment.

The new MR-LST-AR model is a useful model to describe the rater consistency of stability and change. In this application, the mutual attachments of parents and emerging adults were related, and this relationship was stronger for the stable elements of attachment than for the short-term changes.

## Data Availability Statement

The raw data supporting the conclusions of this article will be made available by the authors, without undue reservation.

## Ethics Statement

The studies involving human participants were reviewed and approved by Ethics Committee of the Freie Universität Berlin. The patients/participants provided their written informed consent to participate in this study.

## Author Contributions

JB, JH, TK, ML, and ME contributed to the conception and design of the study. JB, JH, and TK collected the data. JB, JH, and EU performed the statistical analysis. JB wrote the first draft of the manuscript. JH, ML, and ME wrote sections of the manuscript. All authors contributed to manuscript revision, read, and approved the submitted version.

## Conflict of Interest

The authors declare that the research was conducted in the absence of any commercial or financial relationships that could be construed as a potential conflict of interest.

## References

[B1] Ainsworth M. D. (1989). Attachments beyond infancy. *Am. Psychol.* 44 709–716. 10.1037/0003-066x.44.4.709 2729745

[B2] Allen J. P. Grande L. Tan J. Loeb E. (2018). Parent and peer predictors of change in attachment security from adolescence to adulthood. *Child Dev.* 89 1120–1132. 10.1111/cdev.12840 28569384 PMC5711609

[B3] Arnett J. J. (2000). Emerging adulthood. A theory of development from the late teens through the twenties. *Am. Psychol.* 55 469–480. 10.1037/0003-066X.55.5.469 10842426

[B4] Asendorpf J. B. Banse R. Wilpers S. Neyer F. J. (1997). Beziehungsspezifische bindungsskalen für erwachsene und ihre validierung durch netzwerk- und tagebuchverfahren [Relationship-specific attachment scales for adults and their validation with network and diary procedures]. *Diagnostica* 43 289–313.

[B5] Asendorpf J. B. Wilpers S. (2000). Attachment security and available support: closely linked relationship qualities. *J. Soc. Pers. Relationsh.* 17 115–138. 10.1177/0265407500171006

[B6] Baldwin M. W. Fehr B. (1995). On the instability of attachment style ratings. *Pers. Relationsh.* 2 247–261. 10.1111/j.1475-6811.1995.tb00090.x

[B7] Bartholomew K. Horowitz L. M. (1991). Attachment styles among young adults: a test of a four-category model. *J. Pers. Soc. Psychol.* 61 226–244. 10.1037/0022-3514.61.2.226 1920064

[B8] Bohn J. Holtmann J. Luhmann M. Koch T. Eid M. (2020). Attachment to parents and well-being after high-school graduation: a study using self- and parents ratings. *J. Happiness Stud.* 21 2493–2525. 10.1007/s10902-019-00190-y

[B9] Bowlby J. (1969). *Attachment and Loss: Attachment*, Vol. 1. New York, NY: Basic Books.

[B10] Bowlby J. (1973). *Attachment and Loss: Separation: Anxiety and Anger*, Vol. 2. New York, NY: Basic Books.

[B11] Campbell D. T. Fiske D. W. (1959). Convergent and discriminant validation by the multitrait-multimethod matrix. *Psychol. Bull.* 56 81–105. 10.1037/h0046016 13634291

[B12] Caron A. Lafontaine M.-F. Bureau J.-F. Levesque C. Johnson S. M. (2012). Comparisions of close relationships: an evaluation of relationship quality and patterns of attachment to parents, friends, and romantic partners in young adults. *Can. J. Behav. Sci.* 44 245–256. 10.1037/a0028013

[B13] Cole D. A. Martin N. C. Steiger J. H. (2005). Empirical and conceptual problems with longitudinal trait-state models: introducing a trait-state-occasion model. *Psychol. Methods* 10 3–20. 10.1037/1082-989X.10.1.3 15810866

[B14] Cook W. L. Kenny D. A. (2005). The actor-partner interdependence model: a model of bidirectional effects in developmental studies. *Int. J. Behav. Dev.* 29 101–109. 10.1080/01650250444000405

[B15] Courvoisier D. A. Nussbeck F. W. Eid M. Geiser C. Cole D. A. (2008). Analyzing the convergent and discriminant validity of states and traits: development and applications of multimethod latent state-trait models. *Psychol. Assess.* 20 270–280. 10.1037/a0012812 18778163

[B16] Davila J. Burge D. Hammen C. (1997). Why does attachment style change? *J. Pers. Soc. Psychol.* 73 826–838. 10.1037/0022-3514.73.4.826 9325595

[B17] De Goede I. H. Branje S. van Duin J. VanderValk I. E. Meeus W. (2012). Romantic relationship commitment and its linkages with commitment to parents and friends during adolescence. *Soc. Dev.* 21 425–442. 10.1111/j.1467-9507.2011.00633.x

[B18] Eid M. (2000). A multitrait-multimethod model with minimal assumptions. *Psychometrika* 65 241–261. 10.1007/bf02294377

[B19] Eid M. Courvoisier D. S. Lischetzke T. (2012). “Structural equation modeling of ambulatory assessment data,” in *Handbook of Research Methods for Studying Daily Life*, eds Mehl M. R. Conner T. S. (New York, NY: Guilford Press), 384–406.

[B20] Eid M. Geiser C. Koch T. (2016). Measuring method effects: from traditional to design-oriented approaches. *Curr. Dir. Psychol. Sci.* 25 275–280. 10.1177/0963721416649624

[B21] Eid M. Holtmann J. Santangelo P. Ebner-Priemer U. (2017). On the definition of latent state-trait models with autoregressive effects: insights from LST-R theory. *Eur. J. Psychol. Assess.* 33 285–295. 10.1027/1015-5759/a000435

[B22] Eid M. Kutscher T. (2014). “Statistical models for analyzing stability and change in happiness,” in *Stability of Happiness. Theories and Evidence on Whether Happiness can Change*, eds Sheldon K. M. Lucas R. E. (Amsterdam: Elsevier), 263–297.

[B23] Eid M. Lischetzke T. Nussbeck F. Trierweiler L. (2003). Separating trait effects from trait-specific method effects in multitrait-multimethod models: a multiple-indicator CT-C(M-1) model. *Psychol. Methods* 8 38–60. 10.1037/1082-989x.8.1.38 12741672

[B24] Eid M. Nussbeck F. W. Geiser C. Cole D. A. Gollwitzer M. Lischetzke T. (2008). Structural equation modeling of multitrait-multimethod data: different models for different types of methods. *Psychol. Methods* 13 230–253. 10.1037/a0013219 18778153

[B25] Fox N. A. Kimmerly N. L. Schafer W. D. (1991). Attachment to mother/attachment to father: a meta-analysis. *Child Dev.* 62 210–225. 10.2307/1130716 1827064

[B26] Fraley R. C. (2002). Attachment stability from infancy to adulthood: meta-analysis and dynamic modeling of developmental mechanisms. *Pers. Soc. Psychol. Rev.* 6 123–151. 10.1207/s15327957pspr0602_03 26627889

[B27] Fraley R. C. (2019). Attachment in adulthood: recent developments, emerging debates, and future directions. *Annu. Rev. Psychol.* 70 401–422. 10.1146/annurev-psych-010418-102813 30609910

[B28] Fraley R. C. Hudson N. W. Heffernan M. E. Segal N. (2015). Are adult attachment styles categorical or dimensional? A taxometric analysis of general and relationship-specific attachment orientations. *J. Pers. Soc. Psychol.* 109 354–368. 10.1037/pspp0000027 25559192

[B29] Fraley R. C. Vicary A. M. Brumbaugh C. C. Roisman G. I. (2011). Patterns of stability in adult attachment: an empirical test of two models of continuity and change. *J. Pers. Soc. Psychol.* 101 974–992. 10.1037/a0024150 21707199

[B30] Funder D. C. Ozer D. J. (2019). Evaluating effect size in psychological research: sense and nonsense. *Adv. Methods Pract. Psychol. Sci.* 2 156–168. 10.1177/2515245919847202

[B31] Furmann W. Simon V. A. Shaffer L. Bouchey H. A. (2002). Adolescents’ working model and styles for relationsships with parents, friends, and romantic partners. *Child Dev.* 73 241–255. 10.1111/1467-8624.00403 14717255

[B32] Geiser C. (2008). *Structural Equation Modeling of Multitrait-Multimethod-Multioccasion Data.* Berlin: Erziehungswissenschaft und Psychologie.

[B33] Golish T. D. (2000). Changes in closeness between adult children and their parents: a turning point analysis. *Commun. Rep.* 13 79–97. 10.1080/08934210009367727

[B34] Hiester M. Nordstrom A. Swenson L. M. (2009). Stability and change in parental attachment and adjusment outcomes during the first semester transition to college life. *J. Coll. Stud. Dev.* 50 521–538. 10.1353/csd.0.0089

[B35] Holtmann J. Koch T. Bohn J. Eid M. (2017). Bayesian analysis of longitudinal multitrait-multimethod data with ordinal response variables. *Br. J. Math. Stat. Psychol.* 70 42–80. 10.1111/bmsp.12081 28116783

[B36] Holtmann J. Koch T. Bohn J. Eid M. (2020). Multimethod assessment of time-stable and time-variable interindividual differences: introduction of a new multitrait-multimethod latent state-trait IRT model. *Eur. J. Psychol. Assess.* 36 1024–1043. 10.1027/1015-5759/a000577

[B37] Jones J. D. Fraley R. C. Ehrlich K. B. Stern J. A. Lejuez C. W. Shaver P. R. (2018). Stability of attachment style in adolescence: an empirical test of alternative developmental processes. *Child Dev.* 89 871–880. 10.1111/cdev.12775 28301042 PMC5600628

[B38] Karreman A. Vingerhoets A. J. (2012). Attachment and well-being: the mediating role of emotion regulation and resilience. *Pers. Individ. Differ.* 53 821–826. 10.1016/j.paid.2012.06.014

[B39] Kirkpatrick L. A. Hazan C. (1994). Attachment styles and close relationships: a four-year prospective study. *Pers. Relationsh.* 1 123–142. 10.1111/j.1475-6811.1994.tb00058.x

[B40] Koch T. (2013). *Multilevel Structural Equation Modeling of Mulitrait-Multimethod-Multioccasion Data.* Unpublished doctoral dissertation. Berlin: Freie Universität Berlin.

[B41] Koch T. Holtmann J. Bohn J. Eid M. (2018). Explaining general and specific factors in longitudinal, multimethod, and bifactor models: some caveats and recommendations. *Psychol. Methods* 23 505–523. 10.1037/met0000146 28737413

[B42] La Guardia J. G. Ryan R. M. Couchman C. E. Deci E. L. (2000). Within-person variantion in security of attachment: a self-determination theory perspective on attachment, need fulfillment, and well-being. *J. Pers. Soc. Psychol.* 79 367–384. 10.1037//0022-3514.79.3.367 10981840

[B43] Little T. D. (2013). *Longitudinal Equation Modeling.* New York, NY: The Guilford Press.

[B44] Lopez F. G. Gormley B. (2002). Stability and change in adult attachment style over the first-year college transition: relations to self-confidence, coping, and distress patterns. *J. Couns. Psychol.* 49 355–364. 10.1037/0022-0167.49.3.355

[B45] Luhmann M. Bohn J. Holtmann J. Koch T. Eid M. (2016). I’m lonely, can’t you tell? Convergent validity of self- and informant ratings of loneliness. *J. Res. Pers.* 61 50–60. 10.1016/j.jrp.2016.02.002

[B46] McArdle J. J. Nesselroade J. R. (2014). *Longitudinal Data Analysis Using Structural Equation Models.* Washington, DC: American Psychological Association.

[B47] Mikulincer M. Shaver P. R. (2016). *Attachment in Adulthood*, 2nd Edn. New York, NY: Guilford Press.

[B48] Muthén L. K. Muthé B. O. (1998-2017). *Mplus User’s Guide*, 8th Edn. Los Angeles, CA: Muthén & Muthén.

[B49] Nussbeck F. Eid M. Geiser C. Courvosier D. Lischetzke T. (2009). A CTC(M-1) model for different types of raters. *Methodol. Eur. J. Res. Methods Behav. Soc. Sci.* 5 88–98. 10.1027/1614-2241.5.3.88

[B50] Pinquart M. Feußner C. Ahnert L. (2013). Meta-analytic evidence for stability in attachments from infancy to early adulthood. *Attach. Hum. Dev.* 15 189–218. 10.1080/14616734.2013.746257 23210665

[B51] Scarpato B. S. Swardfager W. Eid M. Ploubidis G. B. Cogo-Moreira H. (2021). Disentangling trait, occasion-specific, and accumulated situanional effects of psychological distress in adulthood: evidence from the 1958 and 1970 British birth cohorts. *Psychol. Med.* 51 804–814. 10.1017/S0033291719003805 31910922

[B52] Scharfe E. Bartholomew K. (1994). Reliability and stability of adult attachment patterns. *Pers. Relationsh.* 1 23–43. 10.1111/j.1475-6811.1994.tb00053.x

[B53] Scharfe E. Cole V. (2006). Stability and change of attachment representations during emerging adulthood: an examination of mediators and moderators of change. *Pers. Relationsh.* 13 363–374. 10.1111/j.1475-6811.2006.00123.x

[B54] Schermelleh-Engel K. Moosbrugger H. Müller H. (2003). Evaluating the fit of structural equation models: tests of significance and descriptive goodness-of-fit measures. *Methods Psychol. Res. Online* 8 23–74.

[B55] Seiffge-Krenke I. Burk W. J. (2012). Friends or lovers? Person- and variable-oriented perspectives on dyadic similarity in adolescent romantic relationships. *J. Soc. Pers. Relationsh.* 30 711–733. 10.1177/0265407512467562

[B56] Shaver P. R. Brennan K. A. (1992). Attachment styles and the “Big Five” personality traits: their connection with each other and with romantic relationship outcomes. *Pers. Soc. Psychol. Bull.* 18 536–545. 10.1177/0146167292185003

[B57] Steyer R. Mayer A. Geiser C. Cole D. A. (2015). A theory of states and traits – revised. *Annu. Rev. Clin. Psychol.* 11 71–98. 10.1146/annurev-clinpsy-032813-153719 25062476

