# Supplementary material for: Analyzing Stability and Change in Dyadic Attachment: The Multi-Rater Latent State-Trait Model With Autoregressive Effects
Source: Front Psychol. 2021 Jul 1;12:604526. doi: 10.3389/fpsyg.2021.604526 (PMC8280502; doi:10.3389/fpsyg.2021.604526)
Supplement: Supplementary file 1 [file Table_1.pdf]

frontiers

Supplementary Material

Coefficients of the MR-LST-AR Model

Table A1

Mathematical definitions of the coefficients of the MR-LST-AR model.

Coefficient	*Definition*
	Target ratings (k = 1)
Reliability (*Rel*)	$\mathrm{Rel}(Y_{i1l}) = 1 - \frac{\mathrm{Var}(E_{i1l})}{\mathrm{Var}(Y_{i1l})}$
Occasion specificity (*OS*)	$\mathrm{OS}(Y_{i1l}) = \frac{\lambda_{Oj1l}^2 \mathrm{Var}(SR_l)}{\mathrm{Var}(Y_{i1l}) - \mathrm{Var}(E_{i1l})}$
Time consistency (*TCon*) [for l > 1]	$\mathrm{TCon}(Y_{i1l}) = \frac{\lambda_{Ti1l}^2 \mathrm{Var}(T_{i11}) + \lambda_{Oj1l}^2 \beta_{Ol}^2 \mathrm{Var}(O_{l-1})}{\mathrm{Var}(Y_{i1l}) - \mathrm{Var}(E_{i1l})}$
Predictability by trait$_1$ (*Pred*$_{\mathrm{trait1}}$)	$\mathrm{Pred}_{\mathrm{trait1}}(Y_{i1l}) = \frac{\lambda_{\mathrm{T}i1l}^2 \mathrm{Var}(T_{i11})}{\mathrm{Var}(Y_{i1l}) - \mathrm{Var}(E_{i1l})}$
Unpredictability by trait$_1$ (*UPred*$_{\mathrm{trait1}}$) [for l > 1]	$\mathrm{UPred}_{\mathrm{trait1}}(Y_{i1l}) = \frac{\lambda_{\mathrm{O}j1l}^2 \beta_{Ol}^2 \mathrm{Var}(O_{l-1})}{\mathrm{Var}(Y_{i1l}) - \mathrm{Var}(E_{i1l})}$
	Parent ratings (k = 2)
Reliability (*Rel*)	$\mathrm{Rel}(Y_{i2l}) = 1 - \frac{\mathrm{Var}(E_{i2l})}{\mathrm{Var}(Y_{i2l})}$
Occasion specificity (*OS*)	$\mathrm{OS}(Y_{i2l}) = \frac{\lambda_{\mathrm{O}i2l}^2 \mathrm{Var}(SR_l) + \lambda_{\mathrm{OPS}i2l}^2 \mathrm{Var}(SRPS_l)}{\mathrm{Var}(Y_{i2l}) - \mathrm{Var}(E_{i2l})}$
Time consistency (*TCon*) [for l > 1]	$\mathrm{TCon}(Y_{i2l}) = \frac{\lambda_{\mathrm{T}i2l}^2 \mathrm{Var}(T_{i11}) + \lambda_{\mathrm{TPS}i2l}^2 \mathrm{Var}(TPS_{i21}) + \lambda_{\mathrm{O}j2l}^2 \beta_{\mathrm{O}l}^2 \mathrm{Var}(O_{l-1}) + \lambda_{\mathrm{OPS}j2l}^2 \beta_{\mathrm{OPS}l}^2 \mathrm{Var}(OPS_{l-1})}{\mathrm{Var}(Y_{i2l}) - \mathrm{Var}(E_{i2l})}$

Predictability by trait$_1$ (*Pred*$_{\text{trait1}}$)

$$\text{Pred}_{\text{trait1}}(Y_{i2l}) = \frac{\lambda^2_{Ti2l}\text{Var}(T_{i11}) + \lambda^2_{\text{TPS}i2l}\text{Var}(TPS_{i21})}{\text{Var}(Y_{i2l}) - \text{Var}(E_{i2l})}$$

Unpredictability by trait$_1$ (*UPred*$_{\text{trait1}}$) [for *l* > 1]

$$\text{UPred}_{\text{trait1}}(Y_{i2l}) = \frac{\lambda^2_{\text{O}j2l}\beta^2_{\text{O}l}\text{Var}(O_{l-1}) + \lambda^2_{\text{OPS}j2l}\beta^2_{\text{OPS}l}\text{Var}(OPS_{l-1})}{\text{Var}(Y_{i2l}) - \text{Var}(E_{i2l})}$$

Rater consistency (*RCon*)

$$\text{RCon}(Y_{i2l}) = \frac{\lambda^2_{\text{T}i2l}\text{Var}(T_{i11}) + \lambda^2_{\text{O}i2l}\text{Var}(O_l)}{\text{Var}(Y_{i2l}) - \text{Var}(E_{i2l})}$$

Rater specificity (*RS*)

$$\text{RS}(Y_{i2l}) = \frac{\lambda^2_{\text{TPS}i2l}\text{Var}(TPS_{i21}) + \lambda^2_{\text{OPS}i2l}\text{Var}(OPS_l)}{\text{Var}(Y_{i2l}) - \text{Var}(E_{i2l})}$$

Rater-consistent predictability by trait$_1$ (*RConPred*$_{\text{trait1}}$)

$$\text{RConPred}_{\text{trait1}}(Y_{i2l}) = \frac{\lambda^2_{\text{T}i2l}\text{Var}(T_{i11})}{\lambda^2_{\text{T}i2l}\text{Var}(T_{i11}) + \lambda^2_{\text{TPS}i2l}\text{Var}(TPS_{i21})}$$

Rater-specific predictability by trait$_1$ (*RSPred*$_{\text{trait1}}$)

$$\text{RSPred}_{\text{trait1}}(Y_{i1l}) = \frac{\lambda^2_{\text{TPS}i2l}\text{Var}(TPS_{i21})}{\lambda^2_{\text{T}i2l}\text{Var}(T_{i11}) + \lambda^2_{\text{TPS}i2l}\text{Var}(TPS_{i21})}$$

Rater-consistent time consistency (*RConTCon*) [for *l* > 1]

$$\text{RConTCon}(Y_{i1l}) = \frac{\lambda^2_{\text{T}i2l}\text{Var}(T_{i11}) + \lambda^2_{\text{O}i2l}\beta^2_{\text{O}l}\text{Var}(O_{l-1})}{\lambda^2_{\text{T}i2l}\text{Var}(T_{i11}) + \lambda^2_{\text{TPS}i2l}\text{Var}(TPS_{i21}) + \lambda^2_{\text{O}i2l}\beta^2_{\text{O}l}\text{Var}(O_{l-1}) + \lambda^2_{\text{OPS}i2l}\beta^2_{\text{OPS}l}\text{Var}(OPS_{l-1})}$$

Rater-specific time consistency (*RSTCon*) [for *l* > 1]

$$\text{RSTCon}(Y_{i1l}) = \frac{\lambda^2_{\text{TPS}i2l}\text{Var}(TPS_{i21}) + \lambda^2_{\text{OPS}i2l}\beta^2_{\text{OPS}l}\text{Var}(OPS_{l-1})}{\lambda^2_{\text{T}i2l}\text{Var}(T_{i11}) + \lambda^2_{\text{TPS}i2l}\text{Var}(TPS_{i21}) + \lambda^2_{\text{O}i2l}\beta^2_{\text{O}l}\text{Var}(O_{l-1}) + \lambda^2_{\text{OPS}i2l}\beta^2_{\text{OPS}l}\text{Var}(OPS_{l-1})}$$

Rater-consistent occasion specificity (*RConOS*)

$$\text{RConOS}(Y_{i2l}) = \frac{\lambda^2_{\text{O}j2l}\text{Var}(SR_l)}{\lambda^2_{\text{O}j2l}\text{Var}(SR_l) + \lambda^2_{\text{OPS}j2l}\text{Var}(SRPS_l)}$$

Rater-specific occasion specificity (*RSOS*)

$$\text{RSOS}(Y_{i2l}) = \frac{\lambda^2_{\text{OPS}j2l}\text{Var}(SRPS_l)}{\lambda^2_{\text{O}j2l}\text{Var}(SR_l) + \lambda^2_{\text{OPS}j2l}\text{Var}(SRPS_l)}$$

Rater-consistent unpredictability by trait$_1$ (*RConUPred*$_{\text{trait1}}$) [for *l* > 1]	$\text{RConUPred}_{\text{trait1}}(Y_{i2l}) = \frac{\lambda^2_{\text{O}j2l}\beta^2_{\text{O}l}\text{Var}(O_{l-1})}{\lambda^2_{\text{O}j2l}\beta^2_{\text{O}l}\text{Var}(O_{l-1}) + \lambda^2_{\text{OPS}j2l}\beta^2_{\text{OPS}l}\text{Var}(OPS_{l-1})}$
Rater-specific unpredictability by trait$_1$ (*RSUPred*$_{\text{trait1}}$) [for *l* > 1]	$\text{RSUPred}_{\text{trait1}}(Y_{i2l}) = \frac{\lambda^2_{OPSj2l}\beta^2_{OPSl}\text{Var}(OPS_{l-1})}{\lambda^2_{\text{O}j2l}\beta^2_{\text{O}l}\text{Var}(O_{l-1}) + \lambda^2_{\text{OPS}j2l}\beta^2_{\text{OPS}l}\text{Var}(OPS_{l-1})}$

Note. Y_{ikl} with *i*: indicator (1: acceptance; 2: dependability; 3: closeness); *k*: rater (1: target; 2: parent); *l*: measurement occasion;

The coefficients for the target ratings are based on the variance decomposition in Equation 10 to 12. The coefficients for the parent ratings are based on the variance decomposition in Equation 24 These Equations are described in the article. The model can be applied to different sets of indicators and raters and for different numbers of occasions of measurement.

Gender Differences

1 Emerging Adults

To examine gender differences, the LST-AR models were calculated for male and female targets separately. The 574 targets comprise of 379 females and 196 males. The model for the female targets had a good model fit (χ^2 = 86.142 with df = 47; RMSEA = .047; CFI = .984). The results are displayed in Table S1. The model for the male targets had an acceptable model fit (χ^2 = 73.441 with df = 47; RMSEA = .054; CFI = .971). The results are displayed in Table S2.

Table S1
Results of the LST-AR model of female targets' attachment

	a_{ij}	Rel	OS	TCon	Pred	Unpred	$r(S_{i1}, S_{il})$
Y_{11}	4.51	.765	.415	.585	.585		
		[.64;.89]	[.27;.64]	[.36;.73]	[.36;.73]		
Y_{21}	4.49	.644	.348	.652	.652		
		[.50;.77]	[.20;.59]	[.41;.80]	[.41;.80]		
Y_{31}	4.13	.707	.169	.831	.831		
		[.62;.78]	[.08;.31]	[.69;.92]	[.69;.92]		
Y_{12}	4.47	.850	.372	.628	.556	.071	.742
		[.76;.95]	[.23;.53]	[.47;.77]	[.27;.74]	[.01;.24]	[.61;.85]
Y_{22}	4.47	.663	.325	.675	.613	.062	.779
		[.55;.78]	[.18;.49]	[.51;.82]	[.34;.80]	[.01;.22]	[.65;.88]
Y_{32}	4.06	.808	.137	.863	.837	.026	.901
		[.73;.88]	[.06;.49]	[.75;.94]	[.64;.94]	[.00;.12]	[.83;.95]
Y_{13}	4.38	.831	.347	.653	.574	.079	.652
		[.72;.93]	[.21;.51]	[.49;.79]	[.18;.76]	[.01;.36]	[.50;.77]
Y_{23}	4.42	.779	.313	.687	.615	.071	.696
		[.67;.87]	[.17;.48]	[.52;.83]	[.25;.82]	[.00;.33]	[.53;.82]
Y_{33}	4.00	.808	.143	.857	.824	.033	.858
		[.72;.88]	[,06;.26]	[.74;.94]	[.58;.93]	[.00;.18]	[.75;.93]
Y_{14}	4.14	.725	.461	.539	.431	.108	.539
		[.61;.88]	[.29;.60]	[.40;.71]	[.06;.67]	[.01;.46]	[.33;.70]
Y_{24}	4.42	.712	.319	.681	.606	.075	.656
		[.59;.84]	[.17;.49]	[.52;.83]	[.21;.82]	[.01;.38]	[.45;.81]
Y_{34}	4.06	.817	.148	.852	.817	.035	.837
		[.74;.89]	[.07;.25]	[.75;.93]	[.57;.93]	[.00;.22]	[.71;.93]

Note. a_{ij}: intercept; Rel: reliability coefficient; OS: occasion specificity coefficient; TCon: time consistency coefficient; Pred: predictability by trait$_1$ coefficient; Unpred: unpredictability by trait$_1$ coefficient; $r(S_1, S_l)$: measurement error-free correlation between this measurement occasion and the first measurement occasion.
Y_{ij} with *i*: indicator (1: acceptance; 2: dependability; 3: closeness); *l*: measurement occasion; the bootstrapped 95%-confidence intervals in parenthesis.

Table S2

Results of the LST-AR model of male targets' attachment

	a_{ij}	Rel	OS	TCon	Pred	Unpred	$r(S_{i1}, S_{il})$
Y_{11}	4.54	.614	.348	.652	.652		
		[.41;.84]	[.14;.80]	[.20;.86]	[.20;.86]		
Y_{21}	4.45	.767	.596	.404	.404		
		[.51;1.0]	[.35;.99]	[.01;.65]	[.01;.65]		
Y_{31}	3.95	.547	.225	.775	.775		
		[.39;.67]	[.11;.69]	[.31;.89]	[.31;.89]		
Y_{12}	4.52	.698	.260	.740	.704	.035	.789
		[.53;.90]	[.09;.55]	[.45;.91]	[.33;.88]	[.00;.18]	[.55;.93]
Y_{22}	4.41	.730	.569	.431	.354	.077	.592
		[.53;.95]	[.32;.80]	[.20;.68]	[.00;.63]	[.01;.36]	[.37;.77]
Y_{32}	3.90	.765	.158	.842	.820	.021	.867
		[.64;.91]	[.08;.36]	[.64;.92]	[.49;.91]	[.00;.18]	[.71;.93]
Y_{13}	4.50	.639	.323	.677	.627	.050	.685
		[.46;.83]	[.15;.58]	[.42;.85]	[.13;.81]	[.01;.37]	[.39;.86]
Y_{23}	4.44	.872	.468	.532	.459	.072	.502
		[.70;1.0]	[.24;.73]	[.27;.76]	[.00;.72]	[.01;.44]	[.24;.71]
Y_{33}	3.80	.774	.136	.864	.843	.021	.832
		[.65;.90]	[.07;.30]	[.70;.93]	[.51;.92]	[.00;.22]	[.60;.91]
Y_{14}	4.46	.759	.311	.689	.640	.049	.663
		[.60;.95]	[.12;.58]	[.42;.88]	[.16;.86]	[.00;.38]	[.34;.86]
Y_{24}	4.35	.798	.504	.496	.417	.079	.438
		[.60;1.0]	[.25;.73]	[.26;.75]	[.00;.71]	[.01;.49]	[.12;.66]
Y_{34}	3.81	.761	.157	.843	.819	.025	.806
		[.61;.89]	[.08;.43]	[.57;.92]	[.26;.91]	[.00;.37]	[.46;.90]

Note. a_{ij}: intercept; Rel: reliability coefficient; OS: occasion specificity coefficient; TCon: time consistency coefficient; Pred: predictability by trait$_1$ coefficient; Unpred: unpredictability by trait$_1$ coefficient; $r(S_1, S_l)$: measurement error-free correlation between this measurement occasion and the first measurement occasion.
Y_{ij} with *i*: indicator (1: acceptance; 2: dependability; 3: closeness); *l*: measurement occasion; the bootstrapped 95%-confidence intervals in parenthesis.

There were no global differences between the results for male and female sample. For one aspect there is a small trend for a higher time consistency in the male sample, for one other items the trend is the other way around. For dependability, the time consistency and the predictability were slightly higher in the female sample. For acceptance, the time consistency and the predictability were slightly higher in the male sample.

2 Parents

The LST-AR model for the 368 mothers had a good model fit (χ^2 = 72.507 with df = 47; RMSEA = .038; CFI = .980). The results are displayed in Table S3. The 94 fathers were a too small sample for such a complex model. The model fit was poor (χ^2 = 93.846 with df = 47; RMSEA = .103; CFI = .884). Additionally, the model had theta problems, meaning that the residual variance of one item (Y_{31}) was negative. Due to these estimation problems, the results of this model are not trustworthy and are therefore not displayed here.

Table S3

Results of the LST-AR model of mothers' attachment

	a_{ij}	Rel	OS	TCon	Pred	Unpred	$r(S_{i1}, S_{il})$
Y_{11}	4.54	.741	.234	.766	.766		
		[.60;1.0]	[.11;.37]	[.63;.89]	[.63;.89]		
Y_{21}	4.42	.575	.127	.873	.873		
		[.43;.74]	[.06;.24]	[.76;.94]	[.76;.94]		
Y_{31}	4.32	.636	.261	.739	.739		
		[.48;.94]	[.14;.41]	[.59;.86]	[.59;.86]		
Y_{12}	4.54	.690	.298	.702	.693	.009	.775
		[.50;.84]	[.17;.47]	[.53;.84]	[.30;.83]	[.00;.26]	[.64;.88]
Y_{22}	4.52	.499	.215	.785	.778	.007	.853
		[.35;.66]	[.10;.44]	[.56;.90]	[.35;.90]	[.00;.24]	[.72;.92]
Y_{32}	4.34	.675	.300	.700	.691	.009	.763
		[.52;.82]	[.15;.52]	[.48;.85]	[.21;.84]	[.00;.31]	[.64;.85]
Y_{13}	4.56	.612	.338	.662	.652	.011	.715
		[.45;.77]	[.18;.50]	[.50;.82]	[.20;.81]	[.00;.39]	[.54;.84]
Y_{23}	4.54	.617	.156	.844	.839	.005	.860
		[.46;.77]	[.07;.32]	[.68;.93]	[.44;.93]	[.00;.27]	[.72;.93]
Y_{33}	4.32	.717	.268	.732	.724	.008	.739
		[.58;.86]	[.14;.44]	[.55;.86]	[.21;.86]	[.00;.39]	[.57;.85]
Y_{14}	4.60	.656	.342	.658	.647	.011	.705
		[.51;.81]	[.19;.48]	[.52;.81]	[.20;.80]	[.00;.45]	[.51;.82]
Y_{24}	4.54	.627	.160	.840	.835	.005	.854
		[.45;.82]	[.08;.28]	[,73;.92]	[.50;.92]	[.00;.29]	[.71;.92]
Y_{34}	4.37	.703	.285	.715	.706	.009	.723
		[.55;.86]	[.15;.44]	[.56;.85]	[.17;.85]	[.00;.48]	[.49;.84]

Note. a_{ij}: intercept; Rel: reliability coefficient; OS: occasion specificity coefficient; TCon: time consistency coefficient; Pred: predictability by trait$_1$ coefficient; Unpred: unpredictability by trait$_1$ coefficient; $r(S_1, S_l)$: measurement error-free correlation between this measurement occasion and the first measurement occasion.
Y_{ij} with *i*: indicator (1: acceptance; 2: dependability; 3: closeness); *l*: measurement occasion; the bootstrapped 95%-confidence intervals in parenthesis.

The results in the sample of mothers do not differ significantly at any point from those in the overall sample of parents. The missing effects may be due to the small number of fathers in the study relative to mothers. However, it should also be noted that the targets in this study self-selected their participating parent, which may mean that the fathers are not average fathers. Fathers who have no contact with their children or whose relationship was strained were not selected.
